# Genome mining based on transcriptional regulatory networks uncovers a novel locus involved in desferrioxamine biosynthesis

**DOI:** 10.1371/journal.pbio.3003183

**Published:** 2025-06-12

**Authors:** Hannah E. Augustijn, Zachary L. Reitz, Le Zhang, Jeanine A. Boot, Somayah S. Elsayed, Gregory L. Challis, Marnix H. Medema, Gilles P. van Wezel

**Affiliations:** 1 Bioinformatics Group, Wageningen University, Wageningen, The Netherlands; 2 Institute of Biology, Leiden University, Leiden, The Netherlands; 3 Department of Chemistry, University of Warwick, Coventry, United Kingdom; 4 Department of Biochemistry and Molecular Biology, Monash University, Clayton, Victoria, Australia; 5 ARC Centre of Excellence for Innovations in Peptide and Protein Science, Biomedicine Discovery Institute, Monash University, Clayton, Victoria, Australia; 6 Department of Microbial Ecology, Netherlands Institute of Ecology (NIOO-KNAW), Wageningen, The Netherlands; Universitat zu Koln, GERMANY

## Abstract

Bacteria produce a plethora of natural products that are in clinical, agricultural and biotechnological use. Genome mining has uncovered millions of biosynthetic gene clusters (BGCs) that encode their biosynthesis, the vast majority of them lacking a clear product or function. Thus, a major challenge is to predict the bioactivities of the molecules these BGCs specify, and how to elicit their expression. Here, we present an innovative strategy whereby we harness the power of regulatory networks combined with global gene expression patterns to predict BGC functions. Bioinformatic analysis of all genes predicted to be controlled by the iron master regulator DmdR1 combined with co-expression data, led to identification of the novel operon *desJGH* that plays a key role in the biosynthesis of the iron overload drug desferrioxamine (DFO) B in *Streptomyces coelicolor*. Deletion of either *desG* or *desH* strongly reduces the biosynthesis of DFO B, while that of DFO E is enhanced. DesJGH most likely act by changing the balance between the DFO precursors. Our work shows the power of harnessing regulation-based genome mining to functionally prioritize BGCs, accelerating the discovery of novel bioactive molecules.

## Abstract 2

Most biosynthetic gene clusters remain uncharacterized, making it difficult to predict their functions and activate their expression. Using an innovative approach that integrates regulatory network analysis with global gene expression data, this study identifies desJGH, a novel operon essential for biosynthesis of the iron overload drug desferrioxamine B in Streptomyces coelicolor.

## Introduction

Within the genetic blueprint of microorganisms lies an immense reservoir of chemical potential, which likely constitutes the mechanistic basis for numerous phenotypes and offers a rich source of raw materials for discovery and development of among others antibiotics, anticancer agents, immunosuppressants, crop protection agents, and industrial ingredients [1,2]. Genome mining efforts have led to the identification of millions of biosynthetic gene clusters (BGCs) predicted to encode the biosynthesis of many thousands of natural product scaffolds [3]. However, only an estimated 3% of these specialized metabolites have undergone experimental characterization thus far, leaving a vast amount of untapped chemical diversity yet to be explored [4].

Identifying the diverse roles of specialized metabolites in microbiome interactions is highly challenging, primarily due to the dynamic nature of the host environment and the difficulties in replicating such conditions in laboratory settings. Moreover, while these molecules exhibit a wide range of functions, only a small fraction of metabolites will directly contribute towards microbiome-associated phenotypes such as disease suppression or growth promotion, or have the necessary properties to yield the next generation of crop protection agents, antibiotics, or food additives [5–7]. As a result, there is a pressing need for generalized strategies to predict the functions of specialized metabolites, enabling us to understand their mechanistic roles in inter-organismal interactions and to gauge their usefulness for industrial and clinical applications.

A major aim in current natural product discovery is to identify ways to reduce the genetic space of sequenced BGCs to manageable numbers, to inform scientists on which BGCs to prioritize in the search for novel bioactivity. Historically, scientists have investigated two dimensions, namely the molecular space via high-throughput screening of compound and strain libraries, followed by the genomic space in the 21st century, by investigating BGCs in sequenced genomes, based on the identification of enzyme-coding genes [8]. Perhaps the most advanced strategy for the latter has thus far been target-based genome mining, which uses self-resistance genes inside BGCs as beacons for recognizing the macromolecular targets of their products. However, the presence of recognizable self-resistance genes seems to be limited to a mere 5%–10% of BGCs, necessitating complementary methods to predict the functions of the remaining specialized metabolic diversity [9,10].

We anticipate that an attractive alternative would be regulation-guided approaches, given that the regulatory system plays a pivotal role in the transcription of BGCs. Overexpression or inactivation of cluster-situated regulatory genes have been used to activate their expression [11–13]. For example, targeting BGCs containing *Streptomyces* antibiotic regulatory protein family regulators enabled the discovery of BGCs encoding the biosynthesis of novel antibiotics [14,15]. Also, the Identification of Natural compound Biosynthesis pathways by Exploiting Knowledge of Transcriptional regulation strategy was able to unveil a previously undetectable BGC by identifying regulatory binding sites of the zinc-dependent regulator ZuR [16]. These early successes at the single-gene or single-BGC level indicate that genome-wide analysis of regulatory networks may be even more successful at unveiling BGC functions.

Here, we introduce a computational omics strategy that leverages genome-wide gene regulation information to provide functional predictions of BGCs in microbes. This novel approach connects genome-wide regulatory information derived from transcription factor binding site (TFBS) prediction to gene co-expression networks, thereby associating genes to functions. Genome-wide regulatory analysis of BGCs of *Streptomyces coelicolor* M145 in combination with co-expression patterns unveiled a novel BGC that had escaped detection by current genome mining software tools. Subsequent mutational analysis and metabolic profiling experiments showed that this BGC plays an important role in the biosynthesis of the well-studied siderophore desferrioxamine (DFO) B. These results illustrate the potential of our method to infer BGC function, facilitate the detection and prioritization of novel BGCs and ultimately pave the way for identifying genes responsible for the biosynthesis of novel bioactive molecules.

## Results and discussion

### 1. Identifying functional associations through gene regulatory networks

A major challenge in genome-mining-based drug discovery lies in prioritizing BGCs within the vast unexplored biosynthetic space, and in particular finding novel ways to predict their function. We hypothesized that regulatory networks that control BGC expression might form a new, third, dimension for screening for potential functions, complementing phenotypic and genomic screening. The concept we propose is that if an unknown BGC (or any cluster of genes) is predicted to be controlled by a transcriptional regulator that responds to a known signal and is connected to a specific physiological response, that BGC may functionally relate to known BGCs controlled in a similar manner. From this point onward, when we refer to a BGC’s “function,” we mean the physiological, ecological role or classification of the metabolite(s) specified by a certain BGC as inferred through shared regulatory context.

To develop such a regulation-based genome mining strategy and assess its validity, we chose to focus on the model organism *S. coelicolor* M145. This microbe, belonging to the phylum Actinomycetota, is renowned for its exceptional ability to produce a wide array of bioactive compounds, making it an interesting target for natural product discovery [17–20]. Moreover, it is the bacterial species with currently the largest number of functionally characterized BGCs, with 17 out of its 27 BGCs having been connected to the production of a known metabolite, making it an ideal organism to assess how well regulation connects to function [21]. To investigate the functional relationships between this microbe’s regulatory machinery and specialized metabolite biosynthesis, we investigated the binding of transcription factors (TFs) to their corresponding binding sites (TFBSs). For this purpose, we used the regulatory data of the LogoMotif database [22]. Seventeen precalculated and manually curated position weight matrices (PWMs) associated with TFs in this database were used for genome-wide predictions of 730 TFBSs, using automated computational matching. The resulting matches can be classified as low, medium, or high confidence based on the prediction scores from the PWM pattern matching. The lower bound is set by a *p*-value threshold, which is further refined using an information content score to differentiate medium/higher-confidence matches, following the detection principles outlined in LogoMotif. Low-confidence matches are included as they may represent low-affinity binding sites capable of driving gene expression [23]. However, they may also increase the likelihood of false positives and should therefore be interpreted with caution.

**Fig 1:**
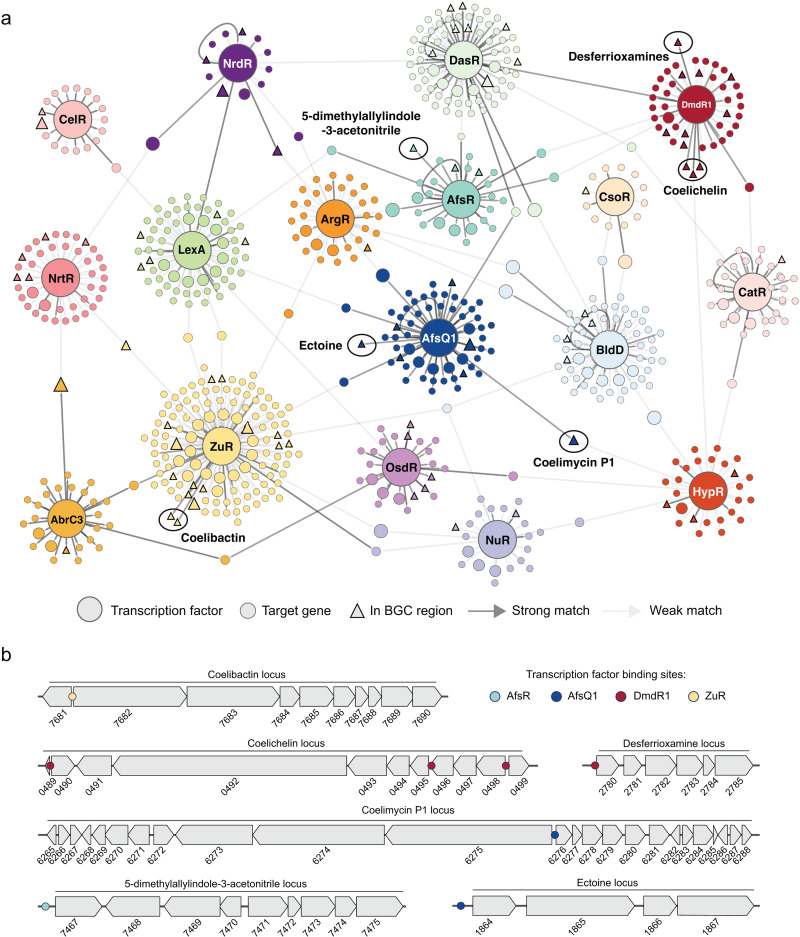
a, Predicted gene regulatory network of *Streptomyces coelicolor* based on 17 well-known regulators. Each node in the network represents a (regulatory) gene, and every edge represents a PWM predicted regulatory interaction between nodes. The edges colored in dark gray indicate strong PWM prediction scores, while the lighter gray shades represent weaker interactions. Matches within BGC regions are depicted as triangles. In six regions (black circled), the matches fall within a co-expressed region, highlighting their functional relation to these compounds. b, Representation of the four co-expressed regions, including the locations of their detected TFBSs as colored dots. All predicted TFBSs have been experimentally validated in pre-existing work. The data underlying this figure can be found at https://zenodo.org/records/15106944.

Based on these predictions, a gene regulatory network was constructed to map out genome-wide regulation of BGCs (Fig 1a). This revealed both direct and indirect regulatory interactions between regulators. Direct regulation, for example, includes the *N*-acetylglucosamine-responsive regulator DasR controlling the iron-dependent regulator DmdR1 [24], and the ribonucleotide reductase-specific repressor NrdR that is predicted to directly regulate the DNA damage response regulator LexA. Indirectly, both DasR and AfsQ1, a regulator of antibiotic biosynthesis and morphological development, appear to control genes involved in transport and phosphorylation. Additionally, AbrC3, a response regulator that transactivates antibiotic production, and OsdR, a regulator of development and stress response, were both predicted to regulate an operon of unknown function, spanning from SCO0173 to SCO0178. Furthermore, AbrC3 and the zinc-responsive regulator ZuR appear to co-regulate the expression of the malic enzyme SCO2951, which plays a role in the production of the antibiotic Actinorhodin [25]. To further target specialized metabolism, TFBSs within BGC regions predicted by antiSMASH were highlighted (Fig 1a). A total of 81 TFBSs were found within antiSMASH BGC regions; 55 of these were at the region peripheries and putatively unrelated to specialized metabolite biosynthesis. To identify which TFBSs were truly linked to biosynthetic pathways, we then refined the boundaries of the BGCs (S1 Table) using literature evidence and gene co-expression patterns (see below). This resulted in the identification of 17 low-confidence and 9 medium/high-confidence BGC-TFBS associations each matching the physiological or ecological functions associated with the corresponding regulon (Fig 1a). These findings agree with existing experimental analyses, thus reinforcing the utility of our approach in accurately identifying BGC-TFBS connections (Fig 1b). For example, there is a clear correlation between TFBSs of the zinc uptake regulator (Zur) and the zinc-regulated coelibactin locus [26], as well as between the pleiotropic antibiotic biosynthesis regulator AfsQ1 and the antibiotic coelimycin P1 [27]. Additionally, we observed a connection between the iron-dependent regulator DmdR1 and the biosynthesis of two iron-chelating compound families that function as siderophores: the DFOs and coelichelin [28,29]. The novel interactions include a regulatory connection between AfsQ1 and the ectoine gene cluster, as well as between AfsR, an antibiotic production regulator, and 5-dimethylallylindole-3-acetonitrile (5-DMAIAN).

### 2. Co-expression analysis and operon-level expansion of the predicted DmdR1 regulon

Next, we aimed to go beyond antiSMASH-detectable BGCs and assess if we could infer the function of any uncharacterized operons and gene clusters using regulatory predictions. Expectedly, the predicted DmdR1 regulon exhibited a clear functional association with siderophores, as evidenced by the connection between its binding sites and known siderophore BGCs [30]. Therefore, we focused on exploring the functional connection between DmdR1 binding sites (*iron boxes*) and iron metabolic genes. A critical issue when using PWMs is the large number of false positive TFBS hits. To address this, we refined the general LogoMotif detection threshold for DmdR1 to be more accurate for *S. coelicolor* by applying the principles previously described for the calibration of the PREDetector algorithm [31]. This approach involves an analysis of the distribution of hits and the ratio of hits in non-coding versus coding regions (S1 Fig). The results demonstrated that higher PWM match scores correlated with a greater frequency of hits detected in non-coding regions, where iron boxes are typically found. By calculating the median score of the non-coding to coding ratio, we established a refined threshold of 22.875, leading to the identification of a total of 39 predicted DmdR1 binding sites (S2 Table). Among these 39 predicted binding sites, we identified 25 unique binding site locations, 22 of which corresponded to previously reported DmdR1 target genes. Based on these predictions, we identified three novel putative DmdR1 target genes: SCO2114, SCO2275, and SCO5998.

**Fig 2:**
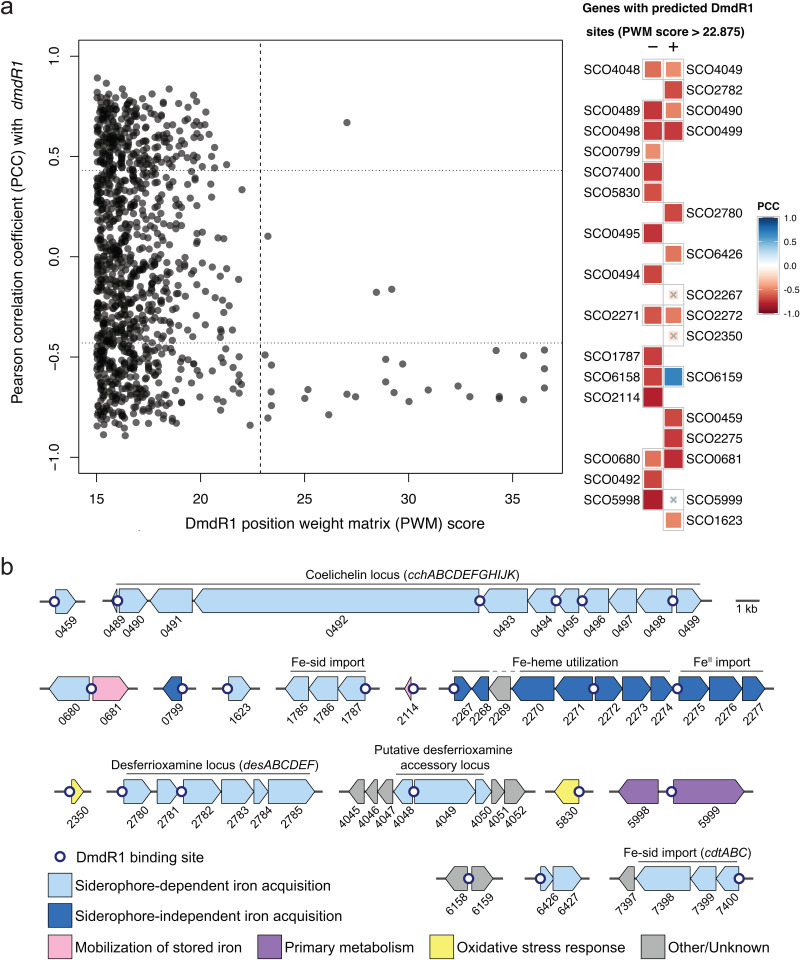
a, Anti-correlation of gene expression between *dmdR1* and its predicted regulon. Left: Pearson correlation coefficients (PCCs) between *dmdR1* and all genes with a DmdR1 position weight matrix (PWM) score greater than 15 in their regulatory region. The vertical dashed line marks the refined PWM score threshold of 22.875. The horizontal dotted lines mark PCC = ±0.43, corresponding to an adjusted *p*-value of 0.05. Right: Target genes immediately downstream of a predicted DmdR1 binding site, ordered by decreasing PWM score. Plus and minus indicate the strand of the target gene. Genes marked with an × did not have significant co-expression with *dmdR1*. Binding site details are given in S2 Table, and the raw data underlying this figure can be found at https://zenodo.org/records/15106944. b, The putative regulon of DmdR1 in *Streptomyces coelicolor* M145. White dots indicate predicted DmdR1 binding sites. Genes are labeled by SCO number and colored by putative function. Clusters are drawn to scale, and arrows represent the direction of transcription.

Bacterial regulons consist not only of genes with TFBSs in their regulatory region, but also any downstream co-operonic genes. DmdR1-controlled operons were predicted using a co-expression analysis of a previously published transcriptome. The RNA-Seq dataset of Lee *and colleagues* [32] was chosen for its relatively high sample count (22 for *S. coelicolor*) and the study’s focus on iron restriction. Reads were retrieved from NCBI SRA and mapped to the *S. coelicolor* M145 genome, and gene count data were processed using previously reported techniques to generate a pairwise gene co-expression matrix [33,34]. Of the 30 predicted DmdR1 target genes with a significant PWM match score, 26 were anti-correlated with transcription of *dmdR1* (Pearson correlation coefficient [PCC] <−0.43, *p* < 0.05, Fig 2a). The co-expression data support the minimum PWM match score of 22.875; below this threshold, no mean anti-correlated expression was identified. Only a single gene with a significant PWM score, the GntR-type regulator SCO6159, was positively co-expressed with *dmdR1* (PCC = 0.69), and the transcription pattern of three putative target genes (SCO2267, SCO2350, SCO5999) did not correlate significantly with that of *dmdR1*, suggesting a more complicated regulation by multiple TFs. DmdR1 target genes were placed into predicted operons using the gene co-expression matrix, as well as strand and intergenic distance, expanding the putative direct regulon of DmdR1 from 25 to 58 genes, which are found across 16 genomic loci (Fig 2b). As expected, DmdR1 binding sites were recovered in the coelichelin and DFO BGCs but not the ZuR-controlled coelibactin BGC, supporting the use of regulatory analysis for linking metallophore BGCs to their corresponding metal. Other logical gene annotations present in the regulon include siderophore-independent iron acquisition, mobilization of stored iron, and oxidative stress response.

### 3. Description of the DmdR1 regulon in S. coelicolor

DmdR1 uses Fe(II) as a co-repressor, and thus the intracellular concentrations of both iron and DmdR1 will affect the expression of the regulon. Although the majority of research on metal-sensitive regulatory proteins focuses on the metal concentration, the anti-co-expression between *dmdR1* and its putative target genes (Fig 2) suggests a considerable role for DmdR1 concentration under the tested conditions [32]. Most of the target genes are more tightly co-expressed with each other (median PCC = 0.90) than anti-co-expressed with *dmdR1* itself (median PCC = –0.66, *p* = 1.2e−8, one-sided Mann–Whitney U test). Intracellular Fe(II) concentrations or other post-transcriptional factors likely decouple the expression of *dmdR1* and its regulon. At least two other repressors directly control the expression of *dmdR1:* DasR (in the absence of glucosamine-6-phosphate) [24], and PhoP (during phosphate starvation) [35]. In the processed RNA-Seq dataset, the expression of *dmdR1* and *dasR* had no linear relationship (PCC = −0.02), while *dmdR1* and *phoP* had anti-correlated expression (PCC = −0.71). However, aspects of post-transcriptional control or not considered, such as by nutrients or posttranslational modification. For example, the DNA binding activity of DasR is controlled by different phosphates, which connects the PhoP and DasR regulons [36].

#### 3.1. Siderophore-mediated iron acquisition.

The majority of the DmdR1 regulon is putatively involved in siderophore pathways. In total, we identified four DmdR1 binding sites in the coelichelin locus (*cch*, SCO0489-0499) and two binding sites in the DFO locus (*desABCD*, SCO2780-2785). After biosynthesis, the ABC transporter complex CchGI (SCO0491 and SCO0493) is presumed to export coelichelin from the cell [30]. The DFO locus does not encode a putative export protein; one of the DmdR1-controlled major facilitator superfamily transporters encoded by SCO0680 or SCO6822 may be responsible. Iron-bound siderophores are transported back into the cell through ABC transporter complexes. The coelichelin locus encodes one complete uptake system, CchCDEF (SCO0494-0497), while the DFO binding protein DesE (SCO2780) is proposed to partner with the incomplete ABC complex encoded by SCO1785-1787. A third, standalone iron-siderophore ABC importer has been characterized, CdtABC (SCO7400-7398) [37,38]. SCO7397, putatively co-operonic with *cdtABC*, is part of the RNase T/ DNA polymerase III family of exonucleases (IPR013520); any relation to iron-starvation is unclear. SCO6426 shows homology to an ABC transporter ATP-binding component; however, ATP-binding residues found in homologs are absent [39]. SCO6426 may be the remnant of an ancestral siderophore ABC transporter system which can still be found complete in other *Streptomyces* species (ex: *S. olivaceus*, KVH11_RS17020–35). After entering the cell, siderophore-bound Fe(III) is reduced by a “siderophore interacting protein” (SIP) that allows for iron release [40]. The DFO locus encodes one SIP reductase, DesF (SCO2781) [30], while putative SIPs SCO0459 and SCO1623 may be involved in iron release from coelichelin and/or xenosiderophores.

#### 3.2. Siderophore-independent iron acquisition.

SCO2267 to SCO2277 are 11 co-expressed genes that contain three predicted DmdR1 binding sites. None of these genes have been characterized, but they are homologous to siderophore-independent iron uptake systems in other organisms. SCO2275-2277 are homologous to *efeO*, *efeB*, and *efeU*, respectively, from an *E. coli* transport system which imports Fe(II) under aerobic, low-pH conditions [41]. A homolog of SCO2268 in *Bordetella bronchiseptica* (BB3589) is adjacent to an unrelated low-pH ferrous iron transport system, *ftrABCD* [42]. However, a BB3589 mutant showed no growth defect, and likewise the exact function of SCO2268 is unclear. SCO2267 and SCO2270-2274 are homologous to *Corynebacterium diptheriae* genes *hmuO*, *htaA*, and *hmuTUV* that are required for acquiring iron from heme and hemoglobin [43–46]. Distant gene SCO0799 encodes a HugZ-like heme oxygenase that may also be involved in Fe-heme utilization [47,48]. SCO2269 is a hypothetical protein that does not match any known protein families; however, its location suggests a role in a siderophore-independent iron acquisition pathway.

#### 3.3. Mobilization of stored iron.

Under iron-replete conditions, *S. coelicolor* stores excess iron as a ferric oxide biomineral within a bacterioferritin nanocage, encoded by SCO2113 [49]. Neighboring SCO2114 has a putative DmdR1 site and encodes a bacterioferritin-associated ferredoxin (Bfd). This family of ferredoxins has been demonstrated to mobilize stored iron for metabolic use by transferring electrons to bacterioferritin for reductive demineralization [49–51]. In turn, SCO0681 encodes a ferredoxin reductase, which could serve as the terminal electron source for Bfd.

#### 3.4. Links to other pathways.

Unchelated intracellular iron catalyzes the formation of reactive oxygen species, linking iron acquisition to oxidative stress responses [52]. SCO5830 encodes a putative thioredoxin-like ferredoxin that was previously found to be highly expressed under copper overload conditions [53], and thus may be involved in a shared response to metal-induced reactive oxygen species. SCO2350 is in the DoxX-like family of membrane proteins, members of which are upregulated during oxidative stress in *Mycobacterium tuberculosis* and *Haemophilus influenzae* [54,55]. SCO6159, GntR-type regulator of the FadR sub-family [56], was the only gene downstream of a putative DmdR1 binding site that was strongly co-expressed with DmdR1 itself (PCC = 0.76). Divergently-transcribed SCO6158 is not a match to any known protein families except for a short C-terminal domain with a [2Fe-2S] binding motif that is identical to that of the siderophore reductase FhuF [57]. This iron-sulfur cluster may be involved in electron transport, like in FhuF itself, or in sensing environmental signals for a regulatory pathway [58]. A DmdR1 binding site between SCO5998 and SCO5999 links DmdR1 to primary metabolism. SCO5998 (MurA2) encodes one of two uridine diphosphate N-acetylglucosamine (UDP-GlcNAc) transferases in *S. coelicolor*, which catalyze the first committed step of peptidoglycan biosynthesis for cell walls [59]. A DasR-mediated link between GlcNAc and DmdR1 has previously been established [24]. SCO5999 encodes aconitase (AcnA), which isomerizes citrate to isocitrate in the citric acid cycle [60]. A secondary role for AcnA has been reported in *E. coli* and *B. subtilis*, and proposed in *S. coelicolor*: under iron starvation or oxidative stress, a [4Fe-4S] cluster disassembles, and AcnA becomes a post-transcriptional regulator of gene expression that binds to mRNA sequences called “iron responsive elements” [60,61]. While SCO5998 expression was anti-correlated with *dmdR1*, SCO5999 expression was uncorrelated.

### 4. Metabolic profiling of an unexplored DmdR1-controlled locus

This systematic mapping of the DmdR1 regulon then provided the opportunity to investigate whether new operons or gene clusters could be identified that would be predicted to specify compounds functioning in iron acquisition. Upon close examination of all individual genes across the regulon, the uncharacterized region from SCO4045 to SCO4052 stood out due to sequence similarity to biosynthetic genes (Fig 2b). The hypothetical protein encoded by SCO4046 is predicted to be intrinsically disordered by MobiDB [62]. SCO4047 is homologous to the HhH-GPD superfamily of base excision repair enzymes [63]. SCO4051 and SCO4052 encode a putative sugar epimerase and dehydrogenase, respectively. Interestingly, SCO4050 encodes a protein similar to the *N*-acyltransferase DesC (encoded by SCO2784), which catalyzes the conversion of *N*-hydroxycadaverine to *N*-hydroxy-*N*-succinylcadaverine (HSC) and *N*-hydroxy-*N*-acetylcadaverine (HAC), the direct precursors of DFO B, in vitro [64]. SCO4048 is a paralog of *desF* (SCO2781), which encodes ferrioxamine reductase. Furthermore, SCO4049 is homologous to genes designated as *desG* in other streptomycetes, and is predicted to encode a penicillin amidase family protein; phylogenetic analysis in Actinomycetota revealed that *desG*, if present, either colocalized with the DFO cluster, or with a separate DmdR1-controlled locus [65]. DesG was originally hypothesized to increase DFO structural diversity by producing phenylacetic acid-capped derivatives in some strains; however, no arylated DFOs have been identified in *S. coelicolor*. Together, SCO4048, SCO4049, and SCO4050 (further referred to as *desJ*, *desG*, and *desH*, respectively) appear to comprise a previously undetected locus, not identified by antiSMASH, putatively related to DFO biosynthesis [65].

To analyze the role of the DmdR1-controlled locus in the production of DFOs, we applied the CRISPR-based editing system (CRISPR-BEST) [66] to construct three knock-out mutants in which either SCO4048 (*desJ*), SCO4049 (*desG*), or SCO4050 (*desH*) had been inactivated. The system allows the introduction of a premature stop codon in the target ORF, thus preventing the production of a functional protein. Using this method, we created null mutants of SCO4048 (*desJ*) with mutations W55* or Q68*, resulting in 186 aa or 173 aa truncation of the gene product, respectively. The introduction of a stop codon at W61 in SCO4049 (*desG*) led to a substantial 721 aa shortening, while mutations W43* or Q91* in SCO4050 (*desH*) resulted in truncations of 163 aa or 115 aa, respectively. PCR followed by DNA sequencing was used to verify the correctness of the knock-out mutants.

**Fig 3:**
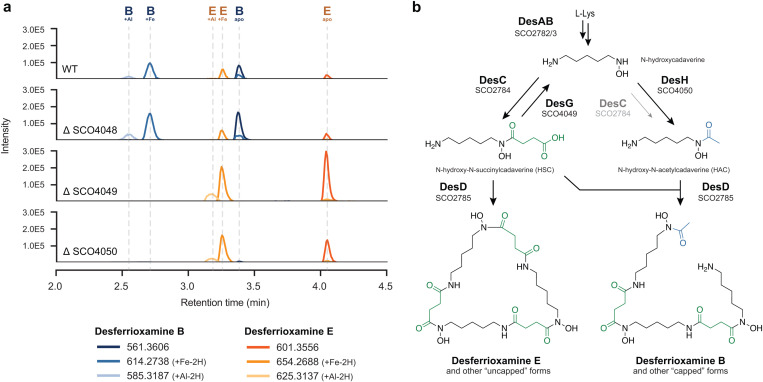
New model for biosynthesis of desferrioxamines B and E. a, Extracted ion chromatograms for m/z values corresponding to DFO-related metabolites in culture extracts of the knock-out mutants of SCO4048 (desJ), SCO4049 (desG), and SCO40450 (desH) compared to the parent *Streptomyces* coelicolor M145 strain. The *desG* mutant fails to produce DFOB, while a 16-fold decrease in DFOB biosynthesis was seen in *desH* mutants (c*f*. S3 Fig). Details on the mass spectrometry data can be found at https://zenodo.org/records/15106944. **b,** Proposed biosynthetic pathway for assembly of desferrioxamines E and B. Main biosynthetic enzymes presented in bold face. DesG and DesH balance intracellular *N*-hydroxy-*N*-succinylcadaverine (HSC) and *N*-hydroxy-*N*-acetylcadaverine (HAC) concentrations by converting HSC to HAC. In the absence of DesG and/or DesH, the cells likely fail to produce sufficient levels of HAC, thereby strongly attenuating the production of DFOB. Although DesC has been shown to be able to catalyze the acetylation of *N*-hydroxycadaverine in vitro, the enzyme can only modestly compensate for the loss of DesH in vivo, underlining the important role played by DesG and DesH in DFOB production (S4 Fig).

To obtain extracts for metabolomics, *S. coelicolor* M145 and its mutant derivatives were grown in a liquid iron-limited medium (ISP-2) for five days. The metabolites produced were adsorbed on Diaion HP20 resin, which was subsequently extracted with methanol and analyzed using liquid chromatography–mass spectrometry (LC–MS), which revealed changes in the production of DFO-related metabolites in each of the mutants compared to the wild-type strain (Fig 3a). The metabolites were annotated by matching the high-resolution mass spectrometry and tandem mass spectrometry (MS/MS) spectra to previously published ones (S2 Fig) [67–69]. Statistical analyses showed that only the levels of DFO B (DFOB) were significantly increased in extracts of the *desJ* mutant as compared to the parental strain (S3 Fig). Metabolomic analysis of ∆*desG* and ∆*desH* revealed an approximate 1000-fold and 16-fold decrease in DFOB production, respectively (Figs 3 and S3). Conversely, the mutants exhibited a significant increase in DFO E (DFOE) and its metal complexes, most likely as a result of the nearly abolished DFOB production.

We genetically complemented the mutants to determine if the effects were due solely to the gene inactivation and not to second-site mutations. For this, constructs were introduced that expressed the respective wild-type genes *desJ, desG* or *desH* from the constitutive *gap* promoter. The complementation constructs were based on vector pSET152 [70], which integrates at the bacteriophage ΦC31 attachment site on the *S. coelicolor* genome. The complemented mutants showed recovery of DFOB production in the complemented strains (S5 Fig). Taken together, our mutational analysis shows that the attenuation of DFOB production in the mutants can be fully explained by the inactivation of *desG* and *desH*.

### 5. Comparative analysis of desferrioxamine biosynthesis across Actinomycetota

DFOB and other capped DFOs have been isolated from many *Streptomyces* strains, as well as several other Actinomycetota. To see if the proposed biosynthetic role for DesGH applies more generally to DFO biosynthesis in other Actinomycetota, we performed a meta-analysis of published DFO producers. In total, we identified reports of DFO production in 46 sequenced strains, comprising mostly *Streptomyces* species (*n* = 34), as well as other Actinomycetota (*n* = 7), Pseudomonadota (*n* = 4), and one member of Bacteroidota (S3 Table). Homologs of d*esG* and *desH* were found in 36 of the genomes, all Actinomycetota. One sequenced DFO producer, *Gordonia rubripertincta* CWB2 was found to produce acetyl-capped DFOs B and A1, but the genome contains only a *desG* homolog without *desH* [71]. A manual inspection of this BGC reveals a distinct GCN5-related acetyltransferase GCWB2_02925 that may be responsible for the acetylated DFOs. The *G. rubripertincta* CWB2 siderophore locus also encodes for mycobactin-like salicylate synthase and non-ribosomal peptide synthetase (NRPS) genes (S7 Table), all of which were upregulated under low-iron conditions [71]. Only one NIS/NRPS hybrid siderophore family has been identified to date, nocardichelins A and B from the unsequenced *Nocardia* strain Acta 3026 [72]. The *G. rubripertincta* CWB2 gene cluster is consistent with the biosynthesis of nocardichelins, as proposed in S8 Fig. Furthermore, nocardichelins were recently detected in extracts from unsequenced *Gordonia* strain Berg02-22.2 [73]. Therefore, the *G. rupripertincta* DFO locus is part of a larger BGC that putatively encodes the biosynthesis of the cryptic nocardichelins, and one of the two other acyltransferase genes in the BGC has presumably replaced *desH*. In all other cases, *desG* and *desH* are putatively co-operonic, and the two genes are fused in *Streptomyces atratus* and *Micrococcus* spp. CH3 and CH7*.* Among collected reports of DFO production, DFOB (Fig 3b) and other acetyl, fatty-acyl, or aryl “capped” DFOs were common, isolated from 34 of 47 sequenced strains. However, in line with our discovery, the nine strains lacking *desGH* exclusively produced DFOE (Fig 3b) and other “uncapped” DFOs with succinylated monomers (S6 Fig).

### 6. Proposed revision of the desferrioxamine biosynthetic pathway

Based on the combination of the above data, we propose the following pathway for DFO biosynthesis in *S. coelicolor*, now including the newly identified desG and desH (Fig 3b). The biosynthesis of DFOE is encoded by the canonical biosynthetic locus *desABCD* (SCO2782-85): DesA and DesB convert l-lysine to *N*-hydroxycadaverine, DesC succinylates *N*-hydroxycadaverine to form HSC [64], and DesD cylcotrimerizes HSC to produce DFOE [74]. In contrast, DesG (SCO4049) and DesH (SCO4050) enable DFOB production (Fig 3). A recent study of DesD concluded that the relative intracellular concentrations of HSC and HAC must be controlled for DFOB formation [74]_._ Previous investigations of DesC in vitro have shown that it is able to catalyze the conversion of *N*-hydroxycadaverine to both HSC and HAC, using succinyl and acetyl-CoA, respectively [64]. However, the relative catalytic efficiency of these two processes has yet to be elucidated. Our experiments strongly suggest that the main function of DesC in vivo is to catalyze the production of HSC, while HAC results primarily from the action of DesH. We propose that DesG, which shows sequence similarity to amidases, de-succinylates HSC to regenerate *N*-hydroxycadaverine, which is then acetylated by the putative acetyltransferase DesH to boost the levels of HAC relative to HSC in high level DFOB producers. Gene fusions of *desGH* observed in some strains are equipped to exploit the high local effective concentration of *N*-hydroxycadaverine generated by the DesG domain, enabling the DesH domain to acetylate *N*-hydroxycadaverine before it can be re-succinylated. The production of DFOB in the ∆*desH* mutant is strongly attenuated but not abolished, consistent with the previously reported ability of DesC to catalyze acylation of *N*-hydroxycadaverine with acetyl-CoA in addition to succinyl-CoA (Fig 3a). Taken together, these data indicate that DesC strongly prefers succinyl-CoA as a substrate over acetyl-CoA, and that DesG and DesH are required to ensure sufficient quantities of HAC are produced to support high level DFOB production in vivo. This biosynthetic model is in line with the available phylogenomic, metabolomic, and genetic evidence, as well as the canonical catalytic chemistry of DesG and DesH homologs.

### 7. Future perspectives

Considering that each Actinobacterial genome may encode up to a thousand TFs, the 17 precalculated TFBS prediction matrices available in this study represent only a small glimpse into the full potential of regulatory genome mining [22,75]. We envision that upcoming high-throughput DNA binding assays, such as DNA Affinity Purification Sequencing (DAP-seq) [76], will significantly expand the available TFBS datasets, greatly increasing the applicability and precision of this approach. In particular, the recently developed multiplexed version, multiDAP [77], offers the possibility to study regulatory conservation across multiple species in parallel, enabling us to assess how well predictions in one species translate to others. Additionally, advancements in peak calling, motif discovery algorithms, and annotations based on gene architecture will further enhance our ability to interpret these datasets [78,79]. By integrating these data with functional annotation approach of this study, paired with gene expression profiles, we anticipate that regulatory genome mining will advance substantially in the near future.

## Conclusions

In conclusion, we have developed a novel computational omics strategy for functional inference of BGCs in microbes, which uses regulatory information to provide clues regarding their functional roles. In this work, we identified novel BGCs involved in siderophore biosynthesis based on regulation by an iron master regulator, but in principle the concept can be extended to any functional class. For example, we anticipate that BGCs that respond to specific environmental signals will be regulated in a similar manner, and such regulatory signatures can thus serve to find BGCs with in a similar ecological role. Uniquely, this method leverages genome-wide gene regulation information derived from TFBS detection combined with gene co-expression network analysis to link biosynthetic genes to their potential functions. A key application of this method is showcased in our study of *S. coelicolor* M145, a well-studied model organism, where we predict the regulons of 17 well-known regulators. Along with the resulting regulatory network, which includes both novel and experimentally validated interactions, we identified nine high-confidence functional associations between known BGCs and regulatory elements. Of these, we selected the iron-dependent repressor DmdR1 and its strong connection to the regulation of siderophore biosynthesis for showcasing the effectiveness of our approach. This analysis, which involved TFBS prediction of the DmdR1 regulon, alongside the detection of co-expression patterns under iron starvation conditions, allowed us to detect an uncharacterized gene cluster with a functional link to iron metabolism. Furthermore, we present evidence that the putative amidase and acyltransferase encoded by *desG* and *desH*, respectively, in this cluster collaborate in the efficient biosynthesis of DFO B by SCO4049 and SCO4050 CRISPR-cBEST knockout mutants and subsequent metabolic profiling experiments. These findings not only validated our hypothesis, but also enabled identification of a novel pathway within the complex biosynthetic route to DFOs. Overall, our results demonstrate the effectiveness of our method in identifying and inferring the function of novel BGCs that escaped detection despite the availability of state-of-the-art genome mining tools. We anticipate that transcriptomics-guided regulatory genome mining, by combining function prediction with application of elicitors that may activate BGCs of interest, will provide pointers as to how to select and activate cryptic BGCs in the extant biosynthetic diversity. This will aid in the identification of their roles in microbiome interactions and guide the discovery of bioactive natural products that are of value for pharmaceutical, agricultural, and biotechnological applications.

## Methods

### Construction of the position weight matrix and sequence motif

Ten previously reported DmdR1 binding sites from *S. coelicolor* were collected from literature [29]. Thereafter, the occurrences of each nucleotide across all positions of the sequences were counted to construct a position frequency matrix (PFM). This PFM was converted to a PWM by applying Bioconductor’s seqLogo v5.29.8 algorithm [80], which calculates the log-likelihood of each nucleotide in the matrix, while taking into account the background nucleotide distributions. Additionally, the information content (IC) of the resulting PWM was calculated using Shannon’s entropy calculation methods. The IC was visualized as a sequence motif with the use of Logomaker v 0.8 [81].

### Identification of DmdR1 binding sites

The genome assembly of *S. coelicolor* A3(2) was downloaded from NCBI using accession GCA_000203835.1. The coding and non-coding regions, as well as the regions spanning from −350 bp to +50 bp relative to the start codons of each gene were extracted with MiniMotif [22] (https://github.com/HAugustijn/MiniMotif). We employed MOODS v1.9.4.1 [82] to query these regions for DmdR1 PWM matches, using a *p*-value threshold of 0.01 and background distribution of 72% representing the GC percentage of *S. coelicolor*. The ratio of hits in non-coding versus coding regions was visualized using the R package ggplot2 [83].

### RNA-Seq data processing and co-expression analyses

*S. coelicolor* A3(2) RNA-Seq data, collected by Lee *and colleagues* [32], was retrieved from the European Nucleotide Archive (PRJEB25075) [84]. Raw read quality was assessed with FastQC [85]. Reads were mapped to the reference genome NC_003888.3 using STAR v2.7.6a [86]: Index files were generated with the parameters “--genomeSAindexNbases 10 --sjdbGTFfeatureExon CDS”, and reads were aligned with the parameter “--alignIntronMax 1”. Mapped reads were indexed using SAMtools v1.3.1 [87] and visualized with the Integrative Genomics Viewer [88]. Per-gene read count tables were generated with featureCounts v2.0.1 [89] using the parameters “-O -M -t CDS -s 2 --fraction”.

The per-gene RNA-Seq count data was further analyzed in R. A minimum gene expression cutoff was applied (≥5 counts in 50% of samples), then counts were normalized by Trimmed Mean of M-values (TMM) and log_2_ transformed using a hyperbolic arcsine pseudocount [90]. A co-expression bias associated with lowly- and highly-expressed genes (of unknown origin, but present in several other RNA-Seq datasets [34]) was mitigated by regressing out the first principal component using the *sva_network* function from the *sva* package (S7 Fig) [33]. The resulting correlation matrix still had an expression-correlated broadening of correlation coefficients, which was corrected by spatial quantile normalization (S7 Fig) [34] and used for further analyses. An all-to-all PCC matrix with corrected two-sided Student *p*-values was calculated using the *corAndPValue* function from the package *WGCNA* [91]. A *p*-value of 0.05 corresponded to a minimum absolute PCC value of 0.43. The correlation matrix was corrected for remaining expression-level-dependent PCC distribution broadening using spatial quantile normalization (*spqn::normalize_correlation*) with the following parameters: ngrp = 20, size_grp = 337, ref_grp = 18 [34]. Subsets of the resulting correlation matrix were used for all downstream analyses.

### Comparative genomics

DFO core loci (*desABCD*) and accessory loci (*desGH*) were found in *Streptomyces* genomes using a modified version of antiSMASH 7 [92] (https://github.com/zreitz/antismash/tree/desGH-7-1). The “desABCD” rule requires matches to all of the following Pfam models with a maximum intergenic distance of 5 kbp: PF00282.22 (*desA*), PF13434.9 (*desB*), PF13523.9 (*desC*), and PF04183.5 (*desD*). The “desGH” rule requires matches to PF01804.21 (*desG*) and PF13523.9 (*desH*) with a maximum intergenic distance of 1 kbp. Genome assemblies for previously reported DFO producers (S3 Table) were downloaded from NCBI Genbank on 21 Nov, 2023, in Genbank format using ncbi-genome-download [93]. The multiSMASH pipeline [94] was used to scan the genomes with antiSMASH and tabulate the results [92]. A gene phylogeny of the resulting desABCD loci was obtained from CORASON, run as part of BiG-SCAPE v1.1.5 using settings “--mix --no-classify --clans-off --cutoffs 1” [95]. The resulting phylogenetic tree was annotated using iTOL v5 [96].

### Bacterial strains and media

*E. coli* strains DH5ɑ and ET12567/pUZ8002 [97] were used for routine cloning and for interspecific conjugation, respectively. *E. coli* transformants were selected on Luria Bertani agar media containing the relevant antibiotics and grown O/N at 37 °C. *S. coelicolor* A3(2) M145 was used as parental strain to construct mutants. All media and routine *Streptomyces* techniques are described in the *Streptomyces* manual [98]. Soy flour mannitol agar plates were used to grow *Streptomyces* strains for preparing spore suspensions.

### Growth conditions and extraction

The cultures were grown in triplicate in 100 mL Erlenmeyer flasks with 1 g of Diaion HP-20 resin (Resindion, Mitsubishi) in 15 mL of International *Streptomyces* Project-2 medium (ISP-2; yeast extract 4 g/L, malt extract 10 g/L, and dextrose 4 g/L at pH 7.2). The medium was inoculated using 1 μL of spore stock and incubated in a rotary shaker at 30 °C. After five days of growth, the resin was vacuum filtered, washed three times with Milli-Q water, and extracted with 3 × 5 mL of methanol. The crude extracts were then dried, weighed, and dissolved in methanol at a final concentration of 1 mg/mL. Media blanks were extracted and prepared in a similar way as negative controls.

### LC–MS-based metabolic profiling

Liquid chromatography-tandem mass spectrometry (LC–MS/MS) acquisition was performed using Shimadzu Nexera X2 ultra high-performance liquid chromatography (UPLC) system, with attached photodiode array detector (PDA), coupled to Shimadzu 9030 QTOF mass spectrometer, equipped with a standard electrospray ionization (ESI) source unit, in which a calibrant delivery system (CDS) is installed. A total of 2 µL of dissolved extracts were injected into a Waters Acquity HSS C18 column (1.8 μm, 100 Å, 2.1 × 100 mm). The column was maintained at 30 °C, and run at a flow rate of 0.5 mL/min, using 0.1% formic acid in H_2_O as solvent A, and 0.1% formic acid in acetonitrile as solvent B. A gradient was employed for chromatographic separation starting at 5% B for 1 min, then 5%–85% B for 9 min, 85%–100% B for 1 min, and finally held at 100% B for 3 min. The column was re-equilibrated to 5% B for 3 min before the next run was started. The LC flow was switched to the waste the first 0.5 min, then to the MS for 13.5 min, then back to the waste to the end of the run.

The MS system was tuned using standard NaI solution (Shimadzu). The same solution was used to calibrate the system before starting. Additionally, a calibrant solution made from ESI tuning mix (Sigma-Aldrich) was introduced through the CDS system, the first 0.5 min of each run, and the masses detected were used for post-run mass correction for the file, ensuring stable accurate mass measurements.

System suitability was checked by regularly measuring a standard sample made of the compounds displayed in Table 1.

**Table 1:** Compounds present in an standard sample used as system suitability check.

Compound	Concentration (μg/mL)	Retention time (min)	Expected *m*/*z*
Paracetamol	25	2,375	152,0712
Caffeine	5	3,246	195,0882
Prednisolone	2,5	5,290	361,2015
Reserpine	1,25	6,186	609,2,812
Clomipramine	1,25	6,379	315,1,628

All the samples were analyzed in positive polarity, using data dependent acquisition mode. In this regard, full scan MS spectra (*m/z* 100–1,700, scan rate 10 Hz, ID enabled) were followed by two data-dependent MS/MS spectra (*m/z* 100–1,700, scan rate 10 Hz, ID disabled) for the two most intense ions per scan. The ions were selected when they reach an intensity threshold of 1,500, isolated at the tuning file Q1 resolution, fragmented using collision-induced dissociation with fixed collision energy (CE 20 eV), and excluded for 1 s before being re-selected for fragmentation. For the ESI source, the parameters were set to interface voltage 4 kV, interface temperature 300 °C, nebulizing gas flow 3 L/min, and drying gas flow 10 L/min. The parameters used for the CDS probe include an interface voltage 4.5 kV, and nebulizing gas flow 1 L/min.

### Comparative metabolomics

Raw LC–MS data were converted to open source mzXML format using LabSolutions software (Shimadzu), and the converted files were imported into MZmine 3.3.0 [99] for data processing. Unless specified otherwise, *m/z* tolerance was set to 0.002 *m/z* or 10.0 ppm, RT tolerance was set to 0.05 min, MS1 noise level was set to 1.0E3, MS2 noise level to 1.0E1, and the minimum absolute height was set to 5.0E2. The option to detect isotope signals below noise level was selected. For feature detection and chromatogram building, the ADAP chromatogram builder [100] was used with positive polarity, centroid mass detector, minimum group size of 5 in number of scans, and a 2.0E3 group intensity threshold. The obtained peaks were smoothed (width: 9), and the chromatograms were deconvoluted using the local minimum search with a 90% chromatographic threshold, 1% minimum relative height, minimum ratio of peak top/edge of 2 and peak duration of 0.03 to 3.00 min. The detected peaks were deisotoped (monotonic shape, maximum charge: 5; representative isotope: most intense). Peak lists from different extracts were aligned (weight for *m/*z: 20, weight for RT: 20, compare isotopic pattern with a minimum score of 50%). The gap filling algorithm was used to detect and fill missing peaks (intensity threshold 1%, RT tolerance: 0.1 min). Duplicate peaks were filtered, and artifacts caused by detector ringing were removed (*m/z* tolerance: 1.0 m/z or 1,000.0 ppm). The aligned peaks were exported to a MetaboAnalyst. From here, peaks were additionally filtered to keep only peaks present in all 3 replicates and not in the media blanks, using in-house scripts. The resulting MetaboAnalyst peak list was uploaded to MetaboAnalyst [101], log transformed, and normalized with Pareto scaling without prior filtering. Missing values were filled with half of the minimum positive value in the original data. Volcano plots were generated using default parameters. Additionally, extracted ion chromatograms have been obtained for the ions of the DFO-related metabolites (*m/z* tolerance 0.001 or 5 ppm, S4 Table). An in-house python script was used to visualize these chromatograms with matplotlib v3.7.2 pyplot [102].

### Plasmids, constructs, and oligonucleotides

All plasmids and constructs described in this work are summarized in S5 Table. The oligonucleotides are listed in S6 Table.

Fragment containing *gapdh* promoter was digested from previously published plasmid pGWS1370 [103] and cloned into pCRISPR-cBEST [66] via the same restriction sites to generated pGWS1384, where the expression of Cas9n (D10A), cytidine deaminase, and uracil-DNA glycosylase inhibitor (UGI) were under the control of *gapdh* promoter instead of *tipA* promoter. Spacers of each targeted gene were selected on CRISPy-web [104] and cloned into NcoI-digested pGWS1384 via single-strand DNA (ssDNA) oligo bridging method. ssDNA oligos SCO4048_W55 and SCO4048_Q68b were used to generate SCO4048 knockout constructs pGWS1582 and pGWS1584, respectively. Similarly, SCO4049 knockout construct pGWS1585 was created using oligo SCO4049_W61. SCO4050 knockout constructs pGWS1598 and pGWS1590 were created employing oligos SCO4050_W43 and SCO4050_Q91, respectively. All the generated knockout constructs were validated by Sanger sequencing using primer sg_T7_R_SnaBI.

For the complementation of SCO4048 null mutant, pGWS1596 was used, an integrative vector based on pSET152 and harboring SCO4048 under the control of *gap* promoter. The *gap* promoter and the entire coding region (+1/+724) of SCO4048 were amplified from *S. coelicolor* M145 genomic DNA using primer pairs Pgap_F and Pgap_R, and SO4048_F and SCO4048_R, respectively. Fragments were cloned into EcoRI and XbaI digested pSET152 via Gibson assembly to generate pGWS1596. Similarly, pGWS1597 and pGWS1598 were created for the complementation of SCO4049 and SCO4050 null mutants, respectively. The coding region (+1/+2,347) of SCO4049 in pGWS1597 was amplified using primers SCO4049_F and SCO4049_R, while the coding region (+1/+619) of SCO4050 in pGWS1598 was amplified using primer pair SCO4050_F and SCO4050_R.

## Supporting information

S1 Fig DmdR1 PWM threshold setting and identification of novel iron boxes in *Streptomyces coelicolor.* Gray violin plot of the distribution of the number of matches to the PWM. The blue S-curve indicates the ratio (in %) of hits in the non-coding versus coding regions of the genome. The orange dotted line is the threshold set by the median score of the hits. The data underlying this Figure can be found at https://zenodo.org/records/15106944.
(PNG)

S2 Fig Measured MS and MS/MS spectra of desferrioxamine (DFO) B and E compared to their respective GNPS library spectra with accessions CCMSLIB00001059066 and CCMSLIB00005467773 of GNPS Mirror Match. Details on the mass spectrometry data can be found at https://zenodo.org/records/15106944.
(PNG)

S3 Fig Volcano plots showing the relative intensities of the mass features detected in the generated knock-out mutants of *Streptomyces coelicolor* in comparison with the wild-type. Mass features were regarded as significantly upregulated (red colored) or downregulated (blue colored) in the knock-out strains, with at least a 2-fold change in intensity and *p*-value ≤0.05. The data underlying this figure can be found in at https://zenodo.org/records/15106944.
(PNG)

S4 Fig Extracted ion chromatograms of DFOB and DFOE related metabolites in the knock-out (Δ) mutants of SCO4048 (*desJ*), SCO4049 (*desG*) and SCO40450 (*desH*) compared to the parent *S. coelicolor* M145 strain. Details on the mass spectrometry data can be found at https://zenodo.org/records/15106944.
(PNG)

S5 Fig Extracted ion chromatograms of DFO-related metabolites in the knock-out (Δ) and complementation (C) mutants of SCO4048 (*desJ*), SCO4049 (*desG*) and SCO40450 (*desH*) compared to the parent *S. coelicolor* M145 strain. Details on the mass spectrometry data can be found at https://zenodo.org/records/15106944.
(PNG)

S6 Fig Midpoint-rooted phylogenetic gene tree of the *desABCD* locus in previously reported DFO producers (S3 Table), produced by CORASON. Green squares indicate that the genome of the strain also contains homologs of *desGH* in addition to *desABCD*, and dark blue squares indicate that the strain was reported to produce acetyl, fatty-acyl, or aryl “capped” DFOs. The genome of *Gordonia rubripertincta* CWB2 contains a homolog of *desG* in an expanded locus. The Newick file can be found at https://zenodo.org/records/15106944.
(PNG)

S7 Fig Mean-expression biases in correlation coefficient distributions after standard processing (top), after regression of the first principal component (middle), and after spatial quantile normalization (bottom). Genes were sorted by mean expression and split into 10 bins of equal size. All-to-all Pearson correlation coefficients were calculated within each bin, and the density was calculated and plotted with the R package *ggridges*. Deviations from a zero-centered distribution (dashed line) suggest non-biological confounders. Solid black lines give the first, second, and third quartiles. The data underlying this figure can be found at https://zenodo.org/records/15106944.
(PNG)

S8 Fig Proposed biosynthesis for nocardichelins and desferrioxamine B in *Gordonia rubripertincta* CWB2. Gene names correspond to S7 Table, while underscores indicate the locus tag prefix “GCWB2_”.
(PNG)

S1 Table Refinement of BGC boundaries through literature evidence and gene co-expression patterns. The following genes, located more than 10 genes from another gene in the cluster, were removed: BGC 6, SCO1195; BGC 13, SCO5826; BGC 25a, SCO7218; BGC 25b, SCO7239.
(PDF)

S2 Table Predicted DmdR1 regulon. (PDF)

S3 Table Overview of DFO producing strains and detected homologs of the *desG* and *desH* genes. (PDF)

S4 Table DFO-related metabolites (*m*/*z* tolerance 0.001 or 5 ppm). (PDF)

S5 Table Overview of the plasmids and constructs. (PDF)

S6 Table Overview of the oligonucleotides. (PDF)

S7 Table Desferrioxamine gene cluster of *Gordonia rubripertincta* CWB2. (PDF)

## Abbreviations

BGCs: biosynthetic gene clusters
DAP-seq: DNA Affinity Purification Sequencing
DFO: desferrioxamine
HAC: *N*-hydroxy-*N*-acetylcadaverine
HSC: succinylcadaverine
IC: information content
LC–MS: liquid chromatography–mass spectrometry
MS/MS: tandem mass spectrometry
NRPS: non-ribosomal peptide synthetase
PCC: Pearson correlation coefficient
PWMs: position weight matrices
ssDNA: single-strand DNA
TFs: transcription factors
TFBS: transcription factor binding site

## References

[1] Wright GD. Unlocking the potential of natural products in drug discovery. Microb Biotechnol. 2019;12(1):55–7. doi: 10.1111/1751-7915.13351 30565871 PMC6302737

[2] Atanasov AG, Zotchev SB, Dirsch VM, International Natural Product Sciences Taskforce, Supuran CT. Natural products in drug discovery: advances and opportunities. Nat Rev Drug Discov. 2021;20(3):200–16. doi: 10.1038/s41573-020-00114-z 33510482 PMC7841765

[3] Tran PN, Yen M-R, Chiang C-Y, Lin H-C, Chen P-Y. Detecting and prioritizing biosynthetic gene clusters for bioactive compounds in bacteria and fungi. Appl Microbiol Biotechnol. 2019;103(8):3277–87. doi: 10.1007/s00253-019-09708-z 30859257 PMC6449301

[4] Gavriilidou A, Kautsar SA, Zaburannyi N, Krug D, Müller R, Medema MH, et al. Compendium of specialized metabolite biosynthetic diversity encoded in bacterial genomes. Nat Microbiol. 2022;7(5):726–35. doi: 10.1038/s41564-022-01110-2 35505244

[5] Beck ML, Song S, Shuster IE, Miharia A, Walker AS. Diversity and taxonomic distribution of bacterial biosynthetic gene clusters predicted to produce compounds with therapeutically relevant bioactivities. J Ind Microbiol Biotechnol. 2023;50(1):kuad024. doi: 10.1093/jimb/kuad024 37653463 PMC10548851

[6] Hibbing ME, Fuqua C, Parsek MR, Peterson SB. Bacterial competition: surviving and thriving in the microbial jungle. Nat Rev Microbiol. 2010;8(1):15–25. 19946288 10.1038/nrmicro2259 PMC2879262

[7] van Bergeijk DA, Terlouw BR, Medema MH, van Wezel GP. Ecology and genomics of Actinobacteria: new concepts for natural product discovery. Nat Rev Microbiol. 2020;18(10):546–58. doi: 10.1038/s41579-020-0379-y 32483324

[8] Chen R, Wong HL, Burns BP. New approaches to detect biosynthetic gene clusters in the environment. Medicines (Basel). 2019;6(1):32. doi: 10.3390/medicines6010032 30823559 PMC6473659

[9] Yan Y, Liu N, Tang Y. Recent developments in self-resistance gene directed natural product discovery. Nat Prod Rep. 2020;37(7):879–92. 31912842 10.1039/c9np00050j PMC7340575

[10] Chen X, Pan H-X, Tang G-L. Newly discovered mechanisms of antibiotic self-resistance with multiple enzymes acting at different locations and stages. Antibiotics (Basel). 2022;12(1):35. doi: 10.3390/antibiotics12010035 36671236 PMC9854587

[11] Lu F, Hou Y, Zhang H, Chu Y, Xia H, Tian Y. Regulatory genes and their roles for improvement of antibiotic biosynthesis in Streptomyces. 3 Biotech. 2017;7(4):250. doi: 10.1007/s13205-017-0875-6 28718097 PMC5513988

[12] van der Heul HU, Bilyk BL, McDowall KJ, Seipke RF, van Wezel GP. Regulation of antibiotic production in Actinobacteria: new perspectives from the post-genomic era. Nat Prod Rep. 2018;35(6):575–604. doi: 10.1039/c8np00012c 29721572

[13] Laureti L, Song L, Huang S, Corre C, Leblond P, Challis GL, et al. Identification of a bioactive 51-membered macrolide complex by activation of a silent polyketide synthase in *Streptomyces ambofaciens*. Proc Natl Acad Sci U S A. 2011;108(15):6258–63. doi: 10.1073/pnas.1019077108 21444795 PMC3076887

[14] Krause J, Handayani I, Blin K, Kulik A, Mast Y. Disclosing the potential of the sarp-type regulator papr2 for the activation of antibiotic gene clusters in streptomycetes. Front Microbiol. 2020;11:225. 32132989 10.3389/fmicb.2020.00225 PMC7040171

[15] Ye S, Molloy B, Pérez-Victoria I, Montero I, Braña AF, Olano C, et al. Uncovering the cryptic gene cluster ahb for 3-amino-4-hydroxybenzoate derived ahbamycins, by searching SARP regulator encoding genes in the *Streptomyces argillaceus* genome. Int J Mol Sci. 2023;24(9):8197. doi: 10.3390/ijms24098197 37175904 PMC10179220

[16] Spohn M, Wohlleben W, Stegmann E. Elucidation of the zinc-dependent regulation in *Amycolatopsis japonicum* enabled the identification of the ethylenediamine-disuccinate ([S,S]-EDDS) genes. Environ Microbiol. 2016;18(4):1249–63. doi: 10.1111/1462-2920.13159 26636770

[17] Ward AC, Allenby NE. Genome mining for the search and discovery of bioactive compounds: the *Streptomyces paradigm*. FEMS Microbiol Lett. 2018;365(24):10.1093/femsle/fny240. doi: 10.1093/femsle/fny240 30265303

[18] Belknap KC, Park CJ, Barth BM, Andam CP. Genome mining of biosynthetic and chemotherapeutic gene clusters in Streptomyces bacteria. Sci Rep. 2020;10(1):1–9. 32029878 10.1038/s41598-020-58904-9 PMC7005152

[19] Nett M, Ikeda H, Moore BS. Genomic basis for natural product biosynthetic diversity in the actinomycetes. Nat Prod Rep. 2009;26(11):1362–84. doi: 10.1039/b817069j 19844637 PMC3063060

[20] Hoskisson PA, van Wezel GP. Streptomyces coelicolor. Trends Microbiol. 2019;27(5):468–9. 30621999 10.1016/j.tim.2018.12.008

[21] Challis G. Exploitation of the *Streptomyces coelicolor* a3(2) genome sequence for discovery of new natural products and biosynthetic pathways. J Ind Microbiol Biotechnol. 2014;41(2):219–32. 24322202 10.1007/s10295-013-1383-2

[22] Augustijn HE, Karapliafis D, Joosten KMM, Rigali S, van Wezel GP, Medema MH. Logomotif: a comprehensive database of transcription factor binding site profiles in Actinobacteria. J Mol Biol. 2024;436(17):168558. 38580076 10.1016/j.jmb.2024.168558

[23] Shahein A, López-Malo M, Istomin I, Olson EJ, Cheng S, Maerkl SJ. Systematic analysis of low-affinity transcription factor binding site clusters in vitro and in vivo establishes their functional relevance. Nat Commun. 2022;13(1):5273. doi: 10.1038/s41467-022-32971-0 36071116 PMC9452512

[24] Craig M, Lambert S, Jourdan S, Tenconi E, Colson S, Maciejewska M, et al. Unsuspected control of siderophore production by N-acetylglucosamine in streptomycetes. Environ Microbiol Rep. 2012;4(5):512–21. doi: 10.1111/j.1758-2229.2012.00354.x 23760896

[25] Rodriguez E, Navone L, Casati P, Gramajo H. Impact of malic enzymes on antibiotic and triacylglycerol production in *Streptomyces coelicolor*. Appl Environ Microbiol. 2012;78(13):4571–9. doi: 10.1128/AEM.00838-12 22544242 PMC3370476

[26] Kallifidas D, Pascoe B, Owen GA, Strain-Damerell CM, Hong H-J, Paget MSB. The zinc-responsive regulator Zur controls expression of the coelibactin gene cluster in *Streptomyces coelicolor*. J Bacteriol. 2010;192(2):608–11. doi: 10.1128/JB.01022-09 19915027 PMC2805322

[27] Wang R, Mast Y, Wang J, Zhang W, Zhao G, Wohlleben W, et al. Identification of two-component system AfsQ1/Q2 regulon and its cross-regulation with GlnR in *Streptomyces coelicolor*. Mol Microbiol. 2013;87(1):30–48. doi: 10.1111/mmi.12080 23106203

[28] Kim Y, Roe J-H, Park J-H, Cho Y-J, Lee K-L. Regulation of iron homeostasis by peroxide-sensitive CatR, a Fur-family regulator in *Streptomyces coelicolor*. J Microbiol. 2021;59(12):1083–91. doi: 10.1007/s12275-021-1457-1 34865197

[29] Flores F, Martín J. Iron-regulatory proteins dmdr1 and dmdr2 of *Streptomyces coelicolor* form two different DNA-protein complexes with iron boxes. Biochem J. 2004;380(2):497–503. 14960152 10.1042/BJ20031945 PMC1224170

[30] Barona-Gómez F, Lautru S, Francou F-X, Leblond P, Pernodet J-L, Challis GL. Multiple biosynthetic and uptake systems mediate siderophore-dependent iron acquisition in *Streptomyces coelicolor* A3(2) and *Streptomyces ambofaciens* ATCC 23877. Microbiology. 2006;152(11):3355–66. 17074905 10.1099/mic.0.29161-0

[31] Hiard S, Marée R, Colson S, Hoskisson P, Titgemeyer F, van Wezel G. Predetector: a new tool to identify regulatory elements in bacterial genomes. Biochem Biophys Res Commun. 2007;357(4):861–4. 17451648 10.1016/j.bbrc.2007.03.180

[32] Lee N, Kim W, Chung J, Lee Y, Cho S, Jang K-S, et al. Iron competition triggers antibiotic biosynthesis in *Streptomyces coelicolor* during coculture with *Myxococcus xanthus*. ISME J. 2020;14(5):1111–24. doi: 10.1038/s41396-020-0594-6 31992858 PMC7174319

[33] Parsana P, Ruberman C, Jaffe AE, Schatz MC, Battle A, Leek JT. Addressing confounding artifacts in reconstruction of gene co-expression networks. Genome Biol. 2019;20(1):94. doi: 10.1186/s13059-019-1700-9 31097038 PMC6521369

[34] Wang Y, Hicks SC, Hansen KD. Co-expression analysis is biased by a mean-correlation relationship [Internet]. bioRxiv. 2020 [cited 2021 Dec 7]. p. 2020.02.13.944777. Available from: https://www.biorxiv.org/content/10.1101/2020.02.13.944777v1

[35] Millan-Oropeza A, Henry C, Lejeune C, David M, Virolle M-J. Expression of genes of the pho regulon is altered in *Streptomyces coelicolor*. Sci Rep. 2020;10(1):8492. 32444655 10.1038/s41598-020-65087-w PMC7244524

[36] Tenconi E, Jourdan S, Motte P, Virolle M-J, Rigali S. Extracellular sugar phosphates are assimilated by Streptomyces in a PhoP-dependent manner. Antonie Van Leeuwenhoek. 2012;102(3):425–33. doi: 10.1007/s10482-012-9763-6 22733060

[37] Bunet R, Brock A, Rexer H-U, Takano E. Identification of genes involved in siderophore transport in *Streptomyces coelicolor* A3(2). FEMS Microbiol Lett. 2006;262(1):57–64. doi: 10.1111/j.1574-6968.2006.00362.x 16907739

[38] Patel P, Song L, Challis G. Distinct extracytoplasmic siderophore binding proteins recognize ferrioxamines and ferricoelichelin in *Streptomyces coelicolor* A3(2). Biochemistry. 2010;49(37):8033–42. 20704181 10.1021/bi100451k

[39] Yu J, Ge J, Heuveling J, Schneider E, Yang M. Structural basis for substrate specificity of an amino acid ABC transporter. Proc Natl Acad Sci U S A. 2015;112(16):5243–8. doi: 10.1073/pnas.1415037112 25848002 PMC4413293

[40] Miethke M, Hou J, Marahiel MA. The siderophore-interacting protein YqjH acts as a ferric reductase in different iron assimilation pathways of *Escherichia coli*. Biochemistry. 2011;50(50):10951–64. doi: 10.1021/bi201517h 22098718

[41] Cao J, Woodhall MR, Alvarez J, Cartron ML, Andrews SC. EfeUOB (YcdNOB) is a tripartite, acid-induced and CpxAR-regulated, low-pH Fe2+ transporter that is cryptic in *Escherichia coli* K-12 but functional in *E. coli* O157:H7. Mol Microbiol. 2007;65(4):857–75. doi: 10.1111/j.1365-2958.2007.05802.x 17627767

[42] Brickman TJ, Armstrong SK. Iron and pH-responsive FtrABCD ferrous iron utilization system of Bordetella species. Mol Microbiol. 2012;86(3):580–93. doi: 10.1111/mmi.12003 22924881 PMC3805130

[43] Schmitt MP. Utilization of host iron sources by *Corynebacterium diphtheriae*: identification of a gene whose product is homologous to eukaryotic heme oxygenases and is required for acquisition of iron from heme and hemoglobin. J Bacteriol. 1997 Feb;179(3):838–45. 9006041 10.1128/jb.179.3.838-845.1997 PMC178768

[44] Wilks A, Schmitt MP. Expression and characterization of a heme oxygenase (hmu o) from *Corynebacterium diphtheriae* iron acquisition requires oxidative cleavage of the heme macrocycle. J Biol Chem. 1998;273(2):837–41. 9422739 10.1074/jbc.273.2.837

[45] Drazek ES, Hammack CA, Schmitt MP. *Corynebacterium diphtheriae* genes required for acquisition of iron from haemin and haemoglobin are homologous to ABC haemin transporters. Mol Microbiol. 2000;36(1):68–84. doi: 10.1046/j.1365-2958.2000.01818.x 10760164

[46] Allen CE, Schmitt MP. Novel hemin binding domains in the *Corynebacterium diphtheriae* HtaA protein interact with hemoglobin and are critical for heme iron utilization by HtaA. J Bacteriol. 2011;193(19):5374–85. doi: 10.1128/JB.05508-11 21803991 PMC3187408

[47] Hu Y, Jiang F, Guo Y, Shen X, Zhang Y, Zhang R. Crystal structure of HugZ, a novel heme oxygenase from *Helicobacter pylori*. J Biol Chem. 2011;286(2):1537–44. 21030596 10.1074/jbc.M110.172007 PMC3020762

[48] Zhang R, Zhang J, Guo G, Mao X, Tong W, Zhang Y, et al. Crystal structure of *Campylobacter jejuni* ChuZ: a split-barrel family heme oxygenase with a novel heme-binding mode. Biochem Biophys Res Commun. 2011;415(1):82–7. doi: 10.1016/j.bbrc.2011.10.016 22020097

[49] Jobichen C, Chong TY, Rattinam R, Basak S, Srinivasan M, Pandey KP. Cryo-em structure of bacterioferritin nanocages provides insight into the bio-mineralization of ferritins. bioRxiv. 2021.

[50] Yao H, Wang Y, Lovell S, Kumar R, Ruvinsky AM, Battaile KP, et al. The structure of the BfrB-Bfd complex reveals protein-protein interactions enabling iron release from bacterioferritin. J Am Chem Soc. 2012;134(32):13470–81. doi: 10.1021/ja305180n 22812654 PMC3428730

[51] Wang Y, Yao H, Cheng Y, Lovell S, Battaile KP, Midaugh CR. Characterization of the bacterioferritin/bacterioferritin associated ferredoxin protein-protein interaction in solution and determination of binding energy hot spots. Biochem. 2015;54(40):6162–75. 26368531 10.1021/acs.biochem.5b00937 PMC4708090

[52] Winterbourn C. Toxicity of iron and hydrogen peroxide: the Fenton reaction. Toxicol Lett. 1995;82–83:969–74. 8597169 10.1016/0378-4274(95)03532-x

[53] Dwarakanath S, Chaplin AK, Hough MA, Rigali S, Vijgenboom E, Worrall JAR. Response to copper stress in *Streptomyces lividans* extends beyond genes under direct control of a copper-sensitive operon repressor protein (CsoR). J Biol Chem. 2012;287(21):17833–47. doi: 10.1074/jbc.M112.352740 22451651 PMC3366776

[54] Nambi S, Long JE, Mishra BB, Baker R, Murphy KC, Olive AJ, et al. The oxidative stress network of *Mycobacterium tuberculosis* reveals coordination between radical detoxification systems. Cell Host Microbe. 2015;17(6):829–37. doi: 10.1016/j.chom.2015.05.008 26067605 PMC4465913

[55] Nasreen M, Fletcher A, Hosmer J, Zhong Q, Essilfie A-T, McEwan AG, et al. The alternative sigma factor RpoE2 is involved in the stress response to hypochlorite and in vivo survival of *Haemophilus influenzae*. Front Microbiol. 2021;12:637213. doi: 10.3389/fmicb.2021.637213 33643271 PMC7907618

[56] Tsypik O, Yushchuk O, Zaburannyi N, Flärdh K, Walker S, Fedorenko V, et al. Transcriptional regulators of GntR family in *Streptomyces coelicolor* A3(2): analysis in silico and in vivo of YtrA subfamily. Folia Microbiol (Praha). 2016;61(3):209–20. doi: 10.1007/s12223-015-0426-7 26433722

[57] Trindade I, Hernandez G, Lebègue E, Barrière F, Cordeiro T, Piccioli M. Conjuring up a ghost: structural and functional characterization of FhuF, a ferric siderophore reductase from *E. coli*. J Biol Inorg Chem. 2021;26(2–3):313–26. 33559753 10.1007/s00775-021-01854-y PMC8068687

[58] Mettert EL, Kiley PJ. Fe-S proteins that regulate gene expression. Biochim Biophys Acta. 2015;1853(6):1284–93. doi: 10.1016/j.bbamcr.2014.11.018 25450978 PMC4390428

[59] Świątek MA, Tenconi E, Rigali S, van Wezel GP. Functional analysis of the N-acetylglucosamine metabolic genes of *Streptomyces coelicolor* and role in control of development and antibiotic production. J Bacteriol. 2012;194(5):1136–44. doi: 10.1128/JB.06370-11 22194457 PMC3294797

[60] Viollier PH, Nguyen KT, Minas W, Folcher M, Dale GE, Thompson CJ. Roles of aconitase in growth, metabolism, and morphological differentiation of *Streptomyces coelicolor*. J Bacteriol. 2001;183(10):3193–203. doi: 10.1128/JB.183.10.3193-3203.2001 11325949 PMC95221

[61] Alén C, Sonenshein AL. *Bacillus subtilis* aconitase is an RNA-binding protein. Proc Natl Acad Sci U S A. 1999;96(18):10412–7. 10468622 10.1073/pnas.96.18.10412 PMC17902

[62] Piovesan D, Necci M, Escobedo N, Monzon AM, Hatos A, Mičetić I. Mobidb: intrinsically disordered proteins in 2021. Nucleic Acids Res. 2021;49(D1):D361-7. 10.1093/nar/gkaa1058 PMC7779018 33237329

[63] Faucher F, Doublié S, Jia Z. 8-oxoguanine DNA glycosylases: one lesion, three subfamilies. Int J Mol Sci. 2012;13(6):6711–29. doi: 10.3390/ijms13066711 22837659 PMC3397491

[64] Ronan JL, Kadi N, McMahon SA, Naismith JH, Alkhalaf LM, Challis GL. Desferrioxamine biosynthesis: diverse hydroxamate assembly by substrate-tolerant acyl transferase DesC. Philos Trans R Soc Lond B Biol Sci. 2018;373(1748):20170068. doi: 10.1098/rstb.2017.0068 29685972 PMC5915712

[65] Cruz-Morales P, Ramos-Aboites HE, Licona-Cassani C, Selem-Mójica N, Mejía-Ponce PM, Souza-Saldívar V, et al. Actinobacteria phylogenomics, selective isolation from an iron oligotrophic environment and siderophore functional characterization, unveil new desferrioxamine traits. FEMS Microbiol Ecol. 2017;93(9):fix086. doi: 10.1093/femsec/fix086 28910965 PMC5812494

[66] Tong Y, Whitford CM, Robertsen HL, Blin K, Jørgensen TS, Klitgaard AK. Highly efficient DSB-free base editing for streptomycetes with CRISPR-BEST. Proc Natl Acad Sci U S A. 2019;116(41):20366–75. 31548381 10.1073/pnas.1913493116 PMC6789908

[67] Han EJ, Lee SR, Hoshino S, Seyedsayamdost MR. Targeted discovery of cryptic metabolites with antiproliferative activity. ACS Chem Biol. 2022;17(11):3121–30. doi: 10.1021/acschembio.2c00588 36228140 PMC10171914

[68] Groenewold GS, Van Stipdonk MJ, Gresham GL, Chien W, Bulleigh K, Howard A. Collision-induced dissociation tandem mass spectrometry of desferrioxamine siderophore complexes from electrospray ionization of UO2(2+), Fe3+ and Ca2+ solutions. J Mass Spectrom. 2004;39(7):752–61. doi: 10.1002/jms.646 15282754

[69] Sidebottom AM, Karty JA, Carlson EE. Accurate mass MS/MS/MS analysis of siderophores ferrioxamine B and E1 by collision-induced dissociation electrospray mass spectrometry. J Am Soc Mass Spectrom. 2015;26(11):1899–902. 26323615 10.1007/s13361-015-1242-7

[70] Bierman M, Logan R, O’Brien K, Seno ET, Rao RN, Schoner BE. Plasmid cloning vectors for the conjugal transfer of DNA from *Escherichia coli* to *Streptomyces* spp. Gene. 1992;116(1):43–9. doi: 10.1016/0378-1119(92)90627-2 1628843

[71] Schwabe R, Senges CHR, Bandow JE, Heine T, Lehmann H, Wiche O, et al. Cultivation dependent formation of siderophores by *Gordonia rubripertincta* CWB2. Microbiol Res. 2020;238:126481. doi: 10.1016/j.micres.2020.126481 32497965

[72] Schneider K, Rose I, Vikineswary S, Jones AL, Goodfellow M, Nicholson G, et al. Nocardichelins A and B, siderophores from Nocardia strain acta 3026. J Nat Prod. 2007;70(6):932–5. doi: 10.1021/np060612i 17536856

[73] Santos JD, Vitorino I, De la Cruz M, Díaz C, Cautain B, Annang F. Bioactivities and extract dereplication of actinomycetales isolated from marine sponges. Front Microbiol. 2019;10:727. 31024503 10.3389/fmicb.2019.00727 PMC6467163

[74] Yang J, Banas VS, Rivera GSM, Wencewicz TA. Siderophore synthetase DesD catalyzes N-to-C condensation in desferrioxamine biosynthesis. ACS Chem Biol. 2023;18(6):1266–70. doi: 10.1021/acschembio.3c00167 37207292

[75] Augustijn HE, Roseboom AM, Medema MH, van Wezel GP. Harnessing regulatory networks in Actinobacteria for natural product discovery. J Ind Microbiol Biotechnol. 2024;51:kuae011. doi: 10.1093/jimb/kuae011 38569653 PMC10996143

[76] O’Malley RC, Huang S-SC, Song L, Lewsey MG, Bartlett A, Nery JR, et al. Cistrome and epicistrome features shape the regulatory DNA landscape. Cell. 2016;165(5):1280–92. doi: 10.1016/j.cell.2016.04.038 27203113 PMC4907330

[77] Baumgart LA, Lee JE, Salamov A, Dilworth DJ, Na H, Mingay M, et al. Persistence and plasticity in bacterial gene regulation. Nat Methods. 2021;18(12):1499–505. doi: 10.1038/s41592-021-01312-2 34824476

[78] Augustijn HE, van Nassauw D, Cernat S, Reitz ZL, van Wezel GP, Medema MH. Regulatory genes as beacons for discovery and prioritization of biosynthetic gene clusters in Streptomyces. Biochemistry [Internet]. 2025 Mar 25; Available from: 10.1021/acs.biochem.4c00711 PMC12224297 40133269

[79] Borges Farias A, Sganzerla Martinez G, Galán-Vásquez E, Nicolás MF, Pérez-Rueda E. Predicting bacterial transcription factor binding sites through machine learning and structural characterization based on DNA duplex stability. Brief Bioinform [Internet]. 2024 Sep 23;25(6). Available from: 10.1093/bib/bbae581 PMC11562833 39541188

[80] Crooks GE, Hon G, Chandonia J-M, Brenner SE. WebLogo: a sequence logo generator. Genome Res. 2004;14(6):1188–90. doi: 10.1101/gr.849004 15173120 PMC419797

[81] Tareen A, Kinney JB. Logomaker: beautiful sequence logos in python. Bioinformatics. 2020;36(7):2272–4. 31821414 10.1093/bioinformatics/btz921 PMC7141850

[82] Korhonen J, Martinmäki P, Pizzi C, Rastas P, Ukkonen E. Moods: fast search for position weight matrix matches in DNA sequences. Bioinformatics. 2009;25(23):3181–2. 19773334 10.1093/bioinformatics/btp554 PMC2778336

[83] Wickham H. ggplot2: elegant graphics for data analysis. Springer Science & Business Media. 2009.

[84] Harrison P, Ahamed A, Aslam R, Alako B, Burgin J, Buso N. The European nucleotide archive in 2020. Nucleic Acids Res. 2021;49(D1):D82-5. 10.1093/nar/gkaa1028 PMC7778925 33175160

[85] Andrews S. Fastqc: a quality control tool for high throughput sequence data. Babraham Bioinformatics. 2010. Available from: http://www.bioinformatics.babraham.ac.uk/projects/fastqc/.

[86] Dobin A, Davis C, Schlesinger F, Drenkow J, Zaleski C, Jha S. Star: ultrafast universal RNA-seq aligner. Bioinformatics. 2013;29(1):15–21. 23104886 10.1093/bioinformatics/bts635 PMC3530905

[87] Li H, Handsaker B, Wysoker A, Fennell T, Ruan J, Homer N. The sequence alignment/map format and samtools. Bioinformatics. 2009;25(16):2078–9. 19505943 10.1093/bioinformatics/btp352 PMC2723002

[88] Thorvaldsdóttir H, Robinson JT, Mesirov JP. Integrative Genomics Viewer (IGV): high-performance genomics data visualization and exploration. Brief Bioinform. 2013;14(2):178–92. doi: 10.1093/bib/bbs017 22517427 PMC3603213

[89] Liao Y, Smyth G, Shi W. Featurecounts: an efficient general purpose program for assigning sequence reads to genomic features. Bioinformatics. 2014;30(7):923–30. 24227677 10.1093/bioinformatics/btt656

[90] Johnson KA, Krishnan A. Robust normalization and transformation techniques for constructing gene coexpression networks from RNA-seq data. Genome Biol. 2022;23(1):1. doi: 10.1186/s13059-021-02568-9 34980209 PMC8721966

[91] Langfelder P, Horvath S. WGCNA: an R package for weighted correlation network analysis. BMC Bioinformatics. 2008;9(1):1–13. 19114008 10.1186/1471-2105-9-559 PMC2631488

[92] Blin K, Shaw S, Augustijn HE, Reitz ZL, Biermann F, Alanjary M, et al. antiSMASH 7.0: new and improved predictions for detection, regulation, chemical structures and visualisation. Nucleic Acids Res [Internet]. 2023 May 4; Available from: 10.1093/nar/gkad344 PMC10320115 37140036

[93] Blin K. ncbi-genome-download. 2023. Available from: https://zenodo.org/record/8192432

[94] Reitz ZL. MultiSMASH. 2023. Available from: https://zenodo.org/record/8276144

[95] Navarro-Muñoz JC, Selem-Mojica N, Mullowney MW, Kautsar SA, Tryon JH, Parkinson EI, et al. A computational framework to explore large-scale biosynthetic diversity. Nat Chem Biol. 2020;16(1):60–8. doi: 10.1038/s41589-019-0400-9 31768033 PMC6917865

[96] Letunic I, Bork P. Interactive tree of life (itol) v5: an online tool for phylogenetic tree display and annotation. Nucleic Acids Res. 2021;49(W1):W293–6. 10.1093/nar/gkab301 PMC8265157 33885785

[97] MacNeil DJ, Gewain KM, Ruby CL, Dezeny G, Gibbons PH, MacNeil T. Analysis of *Streptomyces avermitilis* genes required for avermectin biosynthesis utilizing a novel integration vector. Gene. 1992;111(1):61–8. doi: 10.1016/0378-1119(92)90603-m 1547955

[98] Kieser T. Practical Streptomyces genetics. 2000.

[99] Schmid R, Heuckeroth S, Korf A, Smirnov A, Myers O, Dyrlund TS, et al. Integrative analysis of multimodal mass spectrometry data in MZmine 3. Nat Biotechnol. 2023;41(4):447–9. doi: 10.1038/s41587-023-01690-2 36859716 PMC10496610

[100] Myers O, Sumner S, Li S, Barnes S, Du X. One step forward for reducing false positive and false negative compound identifications from mass spectrometry metabolomics data: new algorithms for constructing extracted ion chromatograms and detecting chromatographic peaks. Anal Chem. 2017;89(17):8696–703. 28752754 10.1021/acs.analchem.7b00947

[101] Lu Y, Pang Z, Xia J. Comprehensive investigation of pathway enrichment methods for functional interpretation of LC-MS global metabolomics data. Brief Bioinform. 2023;24(1):bbac553. doi: 10.1093/bib/bbac553 36572652 PMC9851290

[102] Caswell TA, de Andrade ES, Lee A, Droettboom M, Hoffmann T, Klymak J. Matplotlib/matplotlib: rel: v3.7.2 2023. Available from: https://zenodo.org/record/8118151

[103] Zhang L, Ramijan K, Carrión VJ, van der Aart LT, Willemse J, van Wezel GP, et al. An alternative and conserved cell wall enzyme that can substitute for the lipid II synthase MurG. mBio. 2021;12(2):e03381-20. doi: 10.1128/mBio.03381-20 33824209 PMC8092295

[104] Blin K, Pedersen LE, Weber T, Lee SY. CRISPy-web: An online resource to design sgRNAs for CRISPR applications. Synth Syst Biotechnol. 2016;1(2):118–21. doi: 10.1016/j.synbio.2016.01.003 29062934 PMC5640694

