# Supplementary figures and images for: Genome mining based on transcriptional regulatory networks uncovers a novel locus involved in desferrioxamine biosynthesis
Source: PLoS Biol. 2025 Jun 12;23(6):e3003183. doi: 10.1371/journal.pbio.3003183 (PMC12161575; doi:10.1371/journal.pbio.3003183)

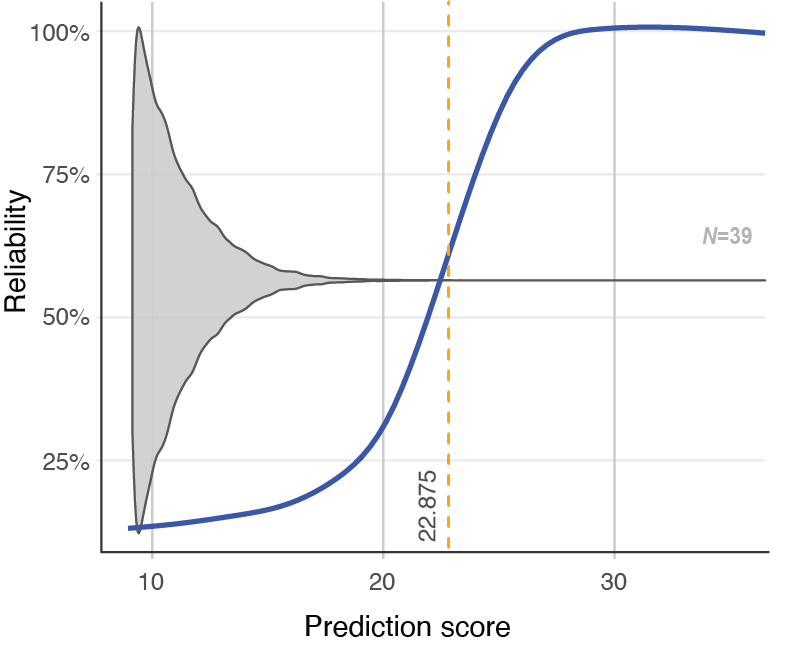

Supplement: S1 Fig — Gray violin plot of the distribution of the number of matches to the PWM. The blue S-curve indicates the ratio (in %) of hits in the non-coding versus coding regions of the genome. The orange dotted line is the threshold set by the median score of the hits. The data underlying this Figure can be found at https://zenodo.org/records/15106944. (PNG) [file pbio.3003183.s001.png]

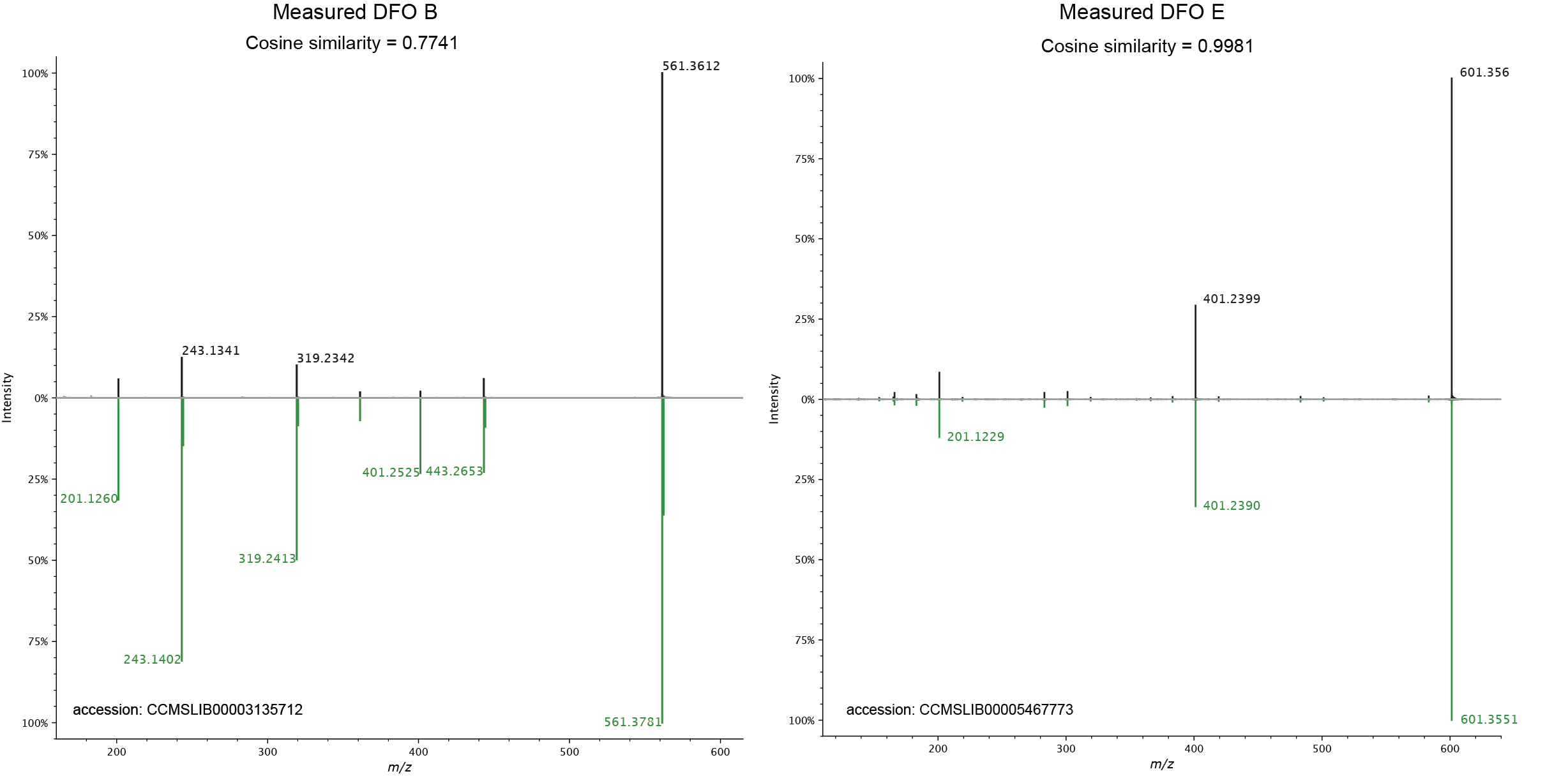

Supplement: S2 Fig — Details on the mass spectrometry data can be found at https://zenodo.org/records/15106944. (PNG) [file pbio.3003183.s002.png]

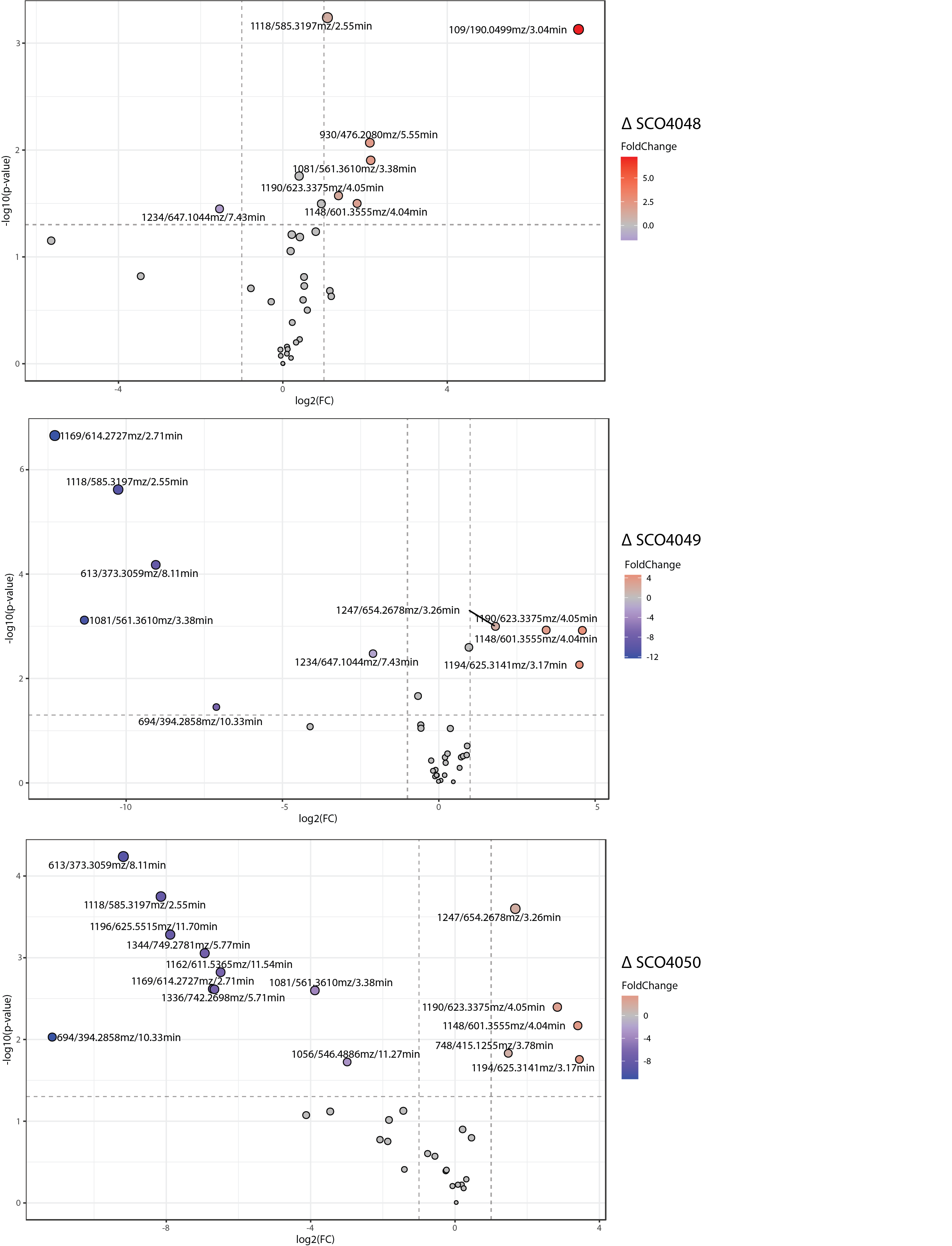

Supplement: S3 Fig — Mass features were regarded as significantly upregulated (red colored) or downregulated (blue colored) in the knock-out strains, with at least a 2-fold change in intensity and p-value ≤0.05. The data underlying this figure can be found in at https://zenodo.org/records/15106944. (PNG) [file pbio.3003183.s003.png]

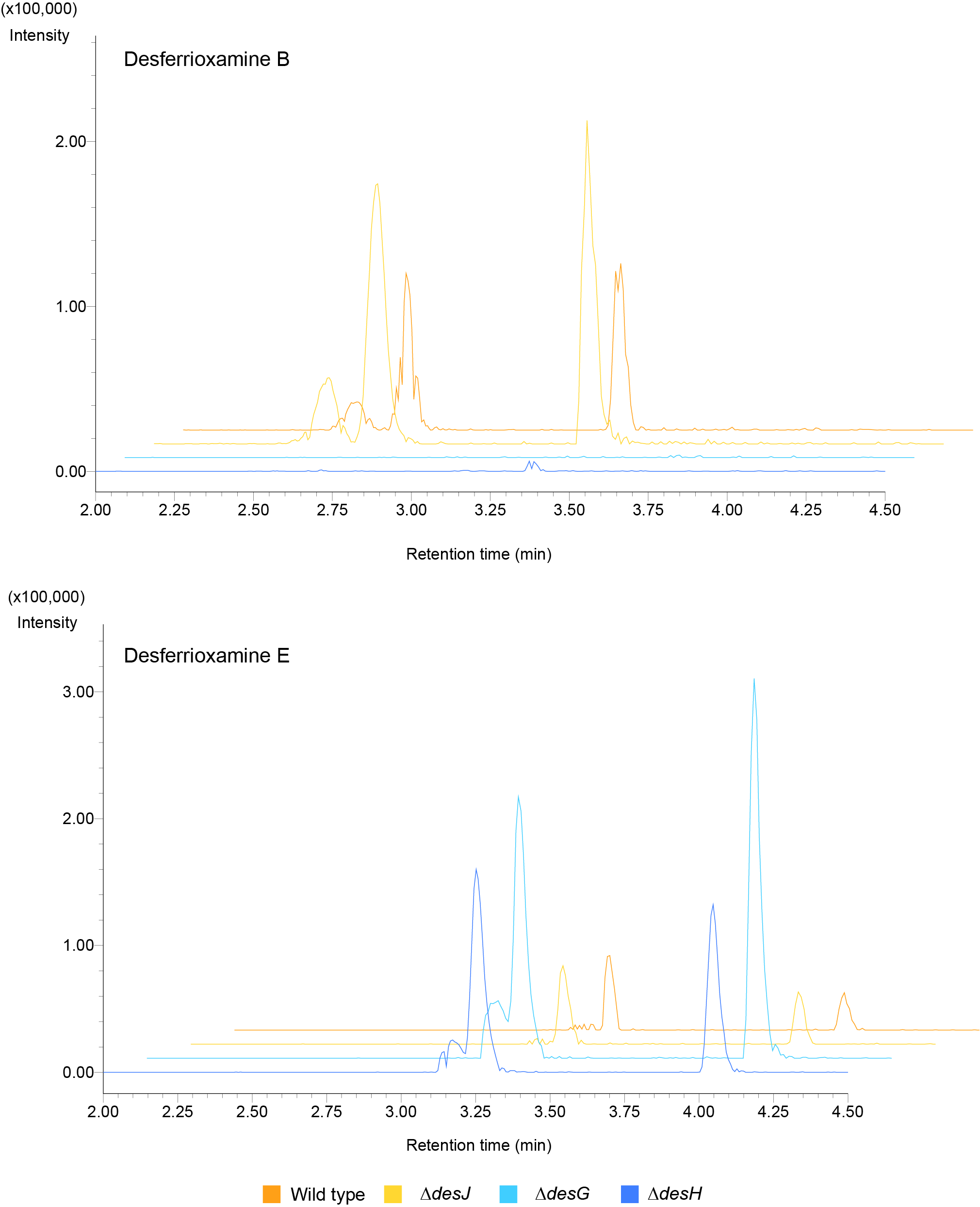

Supplement: S4 Fig — Details on the mass spectrometry data can be found at https://zenodo.org/records/15106944. (PNG) [file pbio.3003183.s004.png]

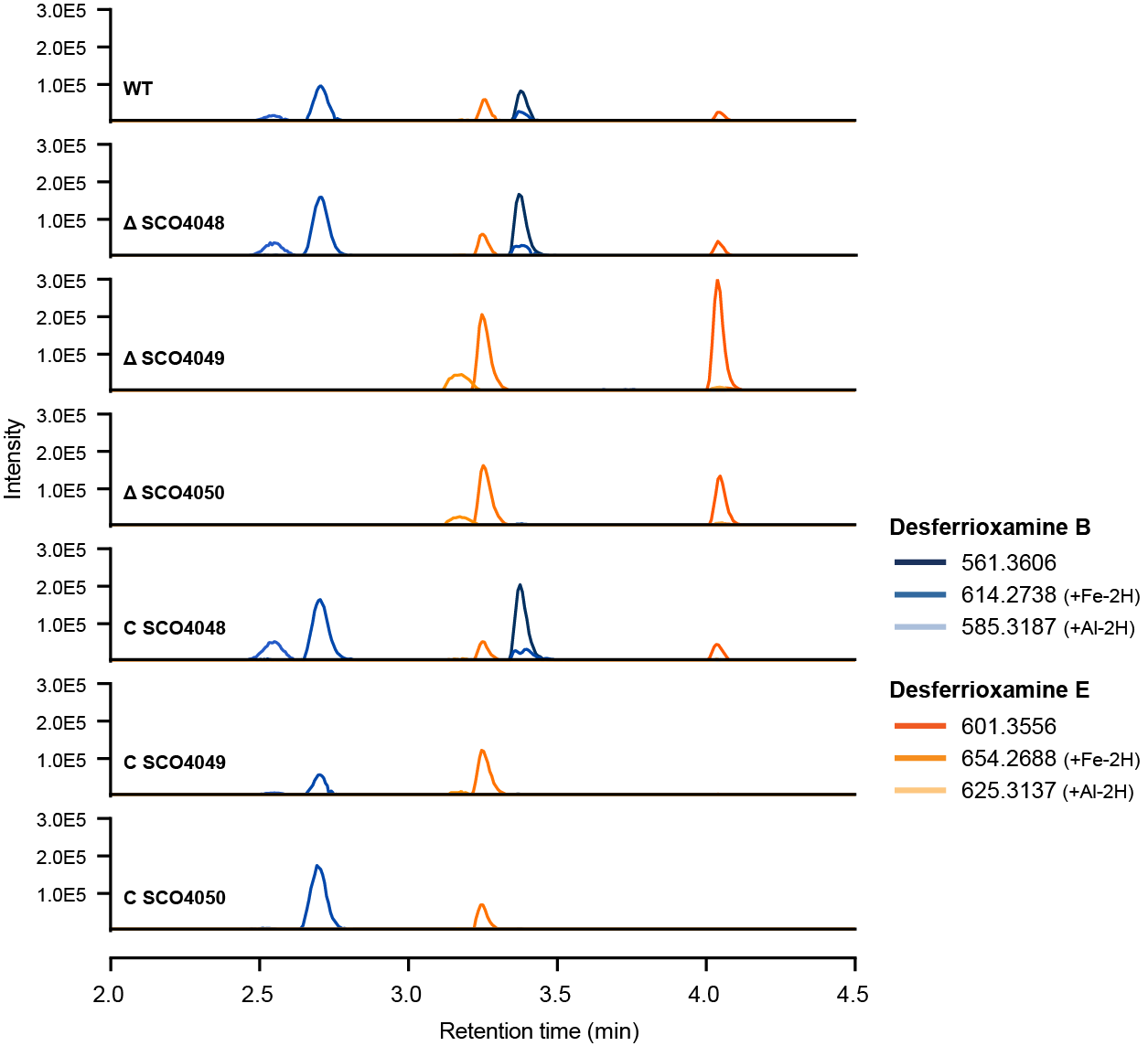

Supplement: S5 Fig — Details on the mass spectrometry data can be found at https://zenodo.org/records/15106944. (PNG) [file pbio.3003183.s005.png]

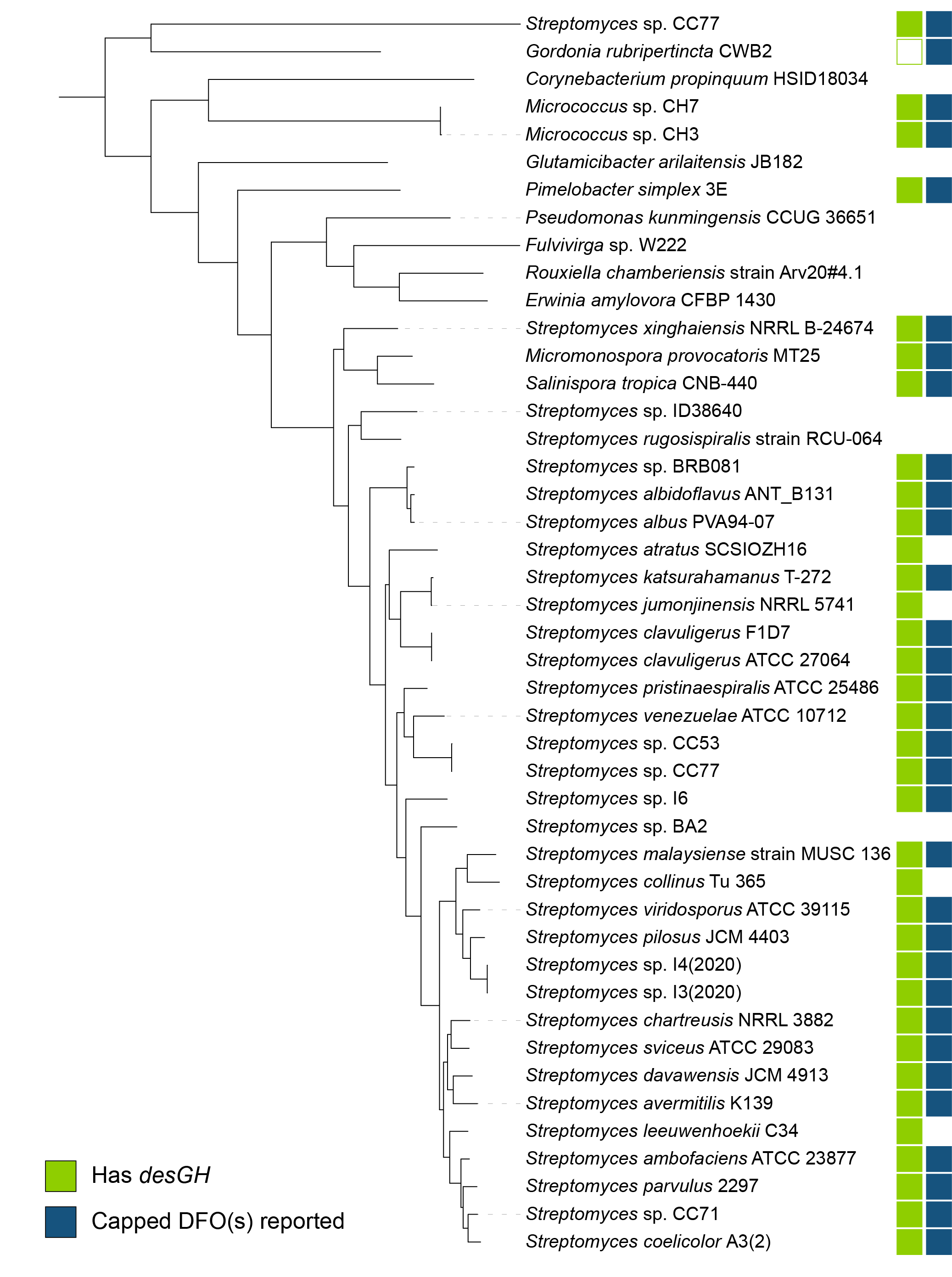

Supplement: S6 Fig — Green squares indicate that the genome of the strain also contains homologs of desGH in addition to desABCD, and dark blue squares indicate that the strain was reported to produce acetyl, fatty-acyl, or aryl “capped” DFOs. The genome of Gordonia rubripertincta CWB2 contains a homolog of desG in an expanded locus. The Newick file can be found at https://zenodo.org/records/15106944. (PNG) [file pbio.3003183.s006.png]

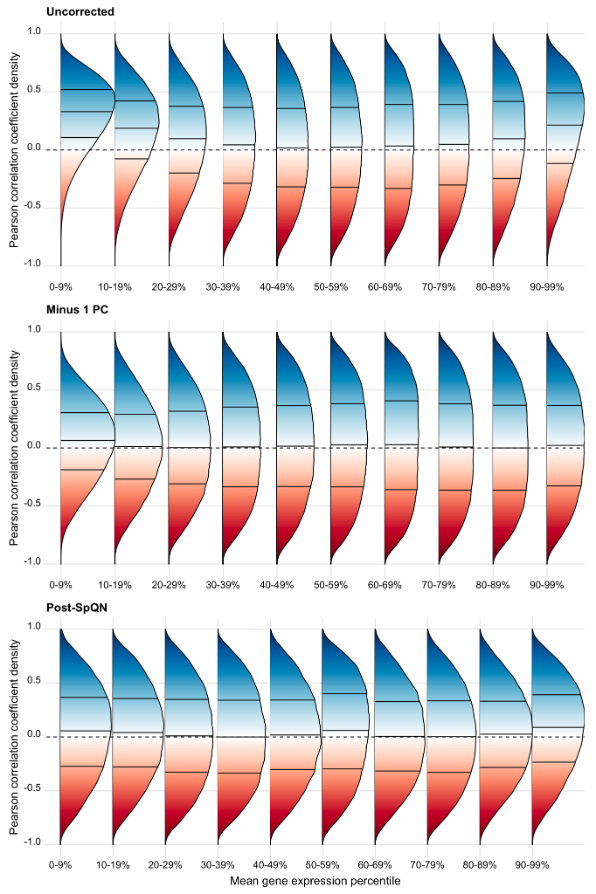

Supplement: S7 Fig — Genes were sorted by mean expression and split into 10 bins of equal size. All-to-all Pearson correlation coefficients were calculated within each bin, and the density was calculated and plotted with the R package ggridges. Deviations from a zero-centered distribution (dashed line) suggest non-biological confounders. Solid black lines give the first, second, and third quartiles. The data underlying this figure can be found at https://zenodo.org/records/15106944. (PNG) [file pbio.3003183.s007.png]

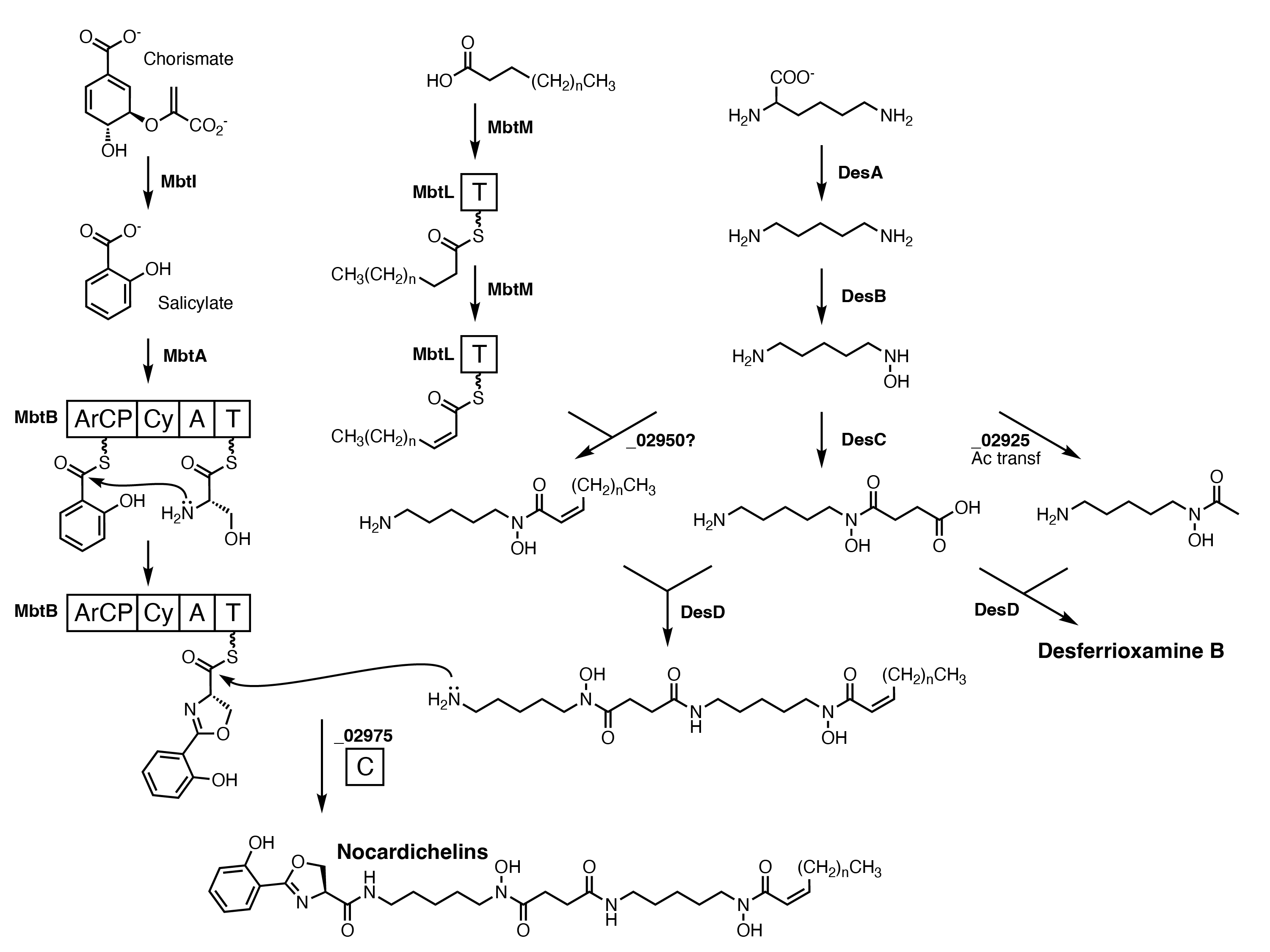

Supplement: S8 Fig — Gene names correspond to S7 Table, while underscores indicate the locus tag prefix “GCWB2_”. (PNG) [file pbio.3003183.s008.png]
