# Supplementary material for: Genome mining based on transcriptional regulatory networks uncovers a novel locus involved in desferrioxamine biosynthesis
Source: PLoS Biol. 2025 Jun 12;23(6):e3003183. doi: 10.1371/journal.pbio.3003183 (PMC12161575; doi:10.1371/journal.pbio.3003183)
Supplement: S1 Table — The following genes, located more than 10 genes from another gene in the cluster, were removed: BGC 6, SCO1195; BGC 13, SCO5826; BGC 25a, SCO7218; BGC 25b, SCO7239. (PDF) [file pbio.3003183.s009.pdf]

Table S1. Refinement of BGC boundaries through literature evidence and gene co-expression patterns. The following genes, located more than 10 genes from another gene in the cluster, were removed: BGC 6, SCO1195; BGC 13, SCO5826; BGC 25a, SCO7218; BGC 25b, SCO7239.

BGC	Product	BGC class	Core genes	Excluded BGCs	Coexpression-based BGC	Literature range	Nucleotide span	Gene span
1	Eicosapentaenoic acid (putative)	T1PKS / hglE-KS	SCO0126, SCO0127	Low variance		SCO0124-0129	86637-139654	SCO0104-SCO0147
2	Carotenoids	terpene	SCO0187, SCO0191		SCO0184-0196	SCO0185-0196	166891-191654	SCO0178-SCO0201
3		lanthipeptide-class-i	SCO0269, SCO0270		SCO0269-0270, SCO0274-0276		246868-270397	SCO0257-SCO0278
4	Coelichelin	NRPS	SCO0492		SCO0489-0499	SCO0489-0499	494260-544087	SCO0473-SCO0508
5		RiPP-like	SCO0753		SCO0753		791701-799942	SCO0750-SCO0755
6	Flaviolins	T3PKS	SCO1206		SCO1205-1206	SCO1206-1208	1258218-1297040	SCO1186-SCO1225
7	Ectoine	ectoine	SCO1866		SCO1864-1867	SCO1864-1867	1995500-2005898	SCO1862-SCO1870
8	Melanin	melanin	SCO2701		SCO2701-2702	SCO2700-2701	2939306-2949875	SCO2694-SCO2706
9	Desferrioxamines	siderophore	SCO2785		SCO2781-2785	SCO2780-2785	3034632-3045603	SCO2781-SCO2789
10	Calcium-dependent antibiotic	NRPS	SCO3230, SCO3231, SCO3232		SCO3215, SCO3217-3222, SCO3227-3249	SCO3210-3249	3524828-3603907	SCO3215-SCO3250
11	Actinorhodin	T2PKS	SCO5087, SCO5088		SCO5070-5092	SCO5071-5092	5496474-5567376	SCO5057-SCO5119
12	Albaflavenone	terpene	SCO5222		SCO5222	SCO5222-5223	5671275-5691836	SCO5212-SCO5231
13	Spore pigment (putative)	T2PKS	SCO5317,SCO5318		SCO5302-5303, SCO5311, SCO5314-5321, SCO5350-5351*	SCO5314-5321	5751945-5824487	SCO5282-SCO5354
14		siderophore	SCO5800		SCO5799-5801		6336091-6346368	SCO5797-SCO5803
15	Undecylprodigiosin	prodigiosin	SCO5887, SCO5889, SCO5890, SCO5891, SCO5892		SCO5877-5880, SCO5882-5899	SCO5877-5899	6430010-6475291	SCO5874-SCO5910
16		RiPP-like	SCO6045		SCO6041-6042,SCO6045		6632343-6643659	SCO6041-SCO6050
17	Geosmin	terpene	SCO6073		SCO6073-6074	SCO6073	6656904-6676224	SCO6065-SCO6080
18		siderophore	SCO6226, SCO6227		SCO6223, SCO6226-6227		6842315-6855522	SCO6221-SCO6231

19	SCB1 / Coelimycin	butyrolactone / T1PKS	SCO6266, SCO6273, SCO6274, SCO6275		SCO6260, SCO6263, SCO6266-6268, SCO6272-6289, SCO6291	SCO6264-6288	6881335-6951537	SCO6259-SCO6291
20		NRPS / thioamide-NRP	SCO6431, SCO6432, SCO6437, SCO6438	Low expression			7088264-7142447	SCO6419-SCO6456
21	SapB	lanthipeptide-class-iii	SCO6681		SCO6681,SCO6685	SCO6681-6685	7409664-7432456	SCO6669-SCO6686
22	Hopene	terpene	SCO6759,SCO6760,SCO6764	Low variance			7506308-7532117	SCO6750-SCO6773
23	Arsono-polyketide	T1PKS	SCO6826,SCO6827		SCO6826-6827,SCO6831	SCO6812-6837	7570412-7618555	SCO6808-SCO6844
24		lanthipeptide-class-i	SCO6928,SCO6929,SCO6930	Low expression			7682907-7709360	SCO6919-SCO6943
25a		other	SCO7190		SCO7188-7190*		7973470-8047403	SCO7176-SCO7239
25b	Germicidin	T3PKS	SCO7221		SCO7220-7221*	SCO7221		
26	5-Dimethylallylindole-3-acetonitrile	indole	SCO7467		SCO7460, SCO7466-7468	SCO7467-7468	8269637-8290764	SCO7455-SCO7476
27a	Coelibactin	T3PKS / NRPS	SCO7671,SCO7682,SCO7683	Low expression		SCO7681-7691	8475102-8548352	SCO7648-SCO7711
27b	2-Methylisoborneol	terpene	SCO7700		SCO7697, SCO7699-7702	SCO7700-7701		
