# Supplementary material for: Genome mining based on transcriptional regulatory networks uncovers a novel locus involved in desferrioxamine biosynthesis
Source: PLoS Biol. 2025 Jun 12;23(6):e3003183. doi: 10.1371/journal.pbio.3003183 (PMC12161575; doi:10.1371/journal.pbio.3003183)
Supplement: S2 Table — (PDF) [file pbio.3003183.s010.pdf]

Table S2. Predicted DmdR1 regulon.

Region	ID	Gene	Annotation	Location	Sequence	First reported by
SCO0459	SCF51A.37		Uncharacterized protein	480954-480972	TTAGGCTAGCCTTGCCTTC	[1]
SCO0489-SCO0490	SCF34.08c		Conserved hypothetical protein	511056-511074	TTAGGTTAGGCTCGCCTAA	[2]
	SCF34.09	*cchJ*	Possible esterase			
SCO0492	SCF34.11c	*cchH*	Probable peptide synthetase	524948-524966	TGAAGGCAGGCTAACCTAA	[1]
SCO0494	SCF34.13c	*cchF*	Probable iron-siderophore binding lipoprotein	527873-527891	TGAGGTAAGCCTACCCTCA	[2]
SCO0495	SCF34.14c	*cchE*	Probable iron-siderophore ABC-transporter ATP-binding protein	528761-528779	CTCGATTAGGCTCACCTTA	[2]
SCO0498	SCF34.17c	*cchB*	Probable peptide monooxygenase	532351-532369	TTAGGTGAGCCTAAGCTAA	[2]
SCO0499	SCF34.18	*cchA*	Possible formyltransferase	532446-532464	TAAGGTAAGCCTTGCTTAA	[2]
SCO0680-SCO0681	SCF15.01c		Possible transmembrane efflux protein	723127-723145	TTAGCTTAGGCTTCCCGGA	[1]
	SCF15.02		Ferredoxin/flavodoxin---nadp+ reductase			
SCO0799	SCF43.10c		Uncharacterized protein	846832-846850	TTAGGTAAGGCTCACCTAA	[2]
SCO1623	SCI41.06		Uncharacterized protein	1737112-1737130	CTAGCTTAGGTATACCTAA	[1]
SCO1787	SCI51.27c		Iron-siderophore uptake system transmembrane protein	1911846-1911864	TTAGGTTAGCCTACCCTGC	[1]
SCO2114	SC6E10.08c		Bacterioferritin-associated ferredoxin	2271956-2271974	TGAGGCAAACCTAACCTTA	This study
SCO2267	SCC75A.13		Heme oxygenase (biliverdin-producing, ferredoxin)	2435898-2435916	TTAGGTAAGCCTAACCATA	[1]
SCO2271	SCC75A.17c		Hypothetical protein	2441256-2441274	AAAGGTAAGGCTAACCTAA	[1]
SCO2272	SCC75A.18		Iron complex transport system substrate-binding protein	2441261-2441279	TAAGGCTAACCTAAGCTCA	[1]
SCO2275	SCC75A.21		Iron uptake system component efeo	2444470-2444488	TTAGGCCAGCCTCACCTTC	This study
SCO2291	SCC75A.37	*axeA*	Acetylxylan esterase	2461315-2461333	GAAGGTGAGCCCGACGTAA	This study
SCO2350	SCC8A.08		Putative oxidoreductase	2520475-2520493	ACTCGTTAGGCTCACCTAA	[2]
SCO2780	SCC105.11	*desE*	Putative secreted protein	3033280-3033298	TGAGGTTAGGCTAACCTAC	[1]
SCO2782	SCC105.13	*desA*	Probable pyridoxal-dependent decarboxylase	3035560-3035577	TTAGGTTAGGCTCACCTAA	[2]

SCO4048-SCO4049	2SCD60.14c		Hypothetical protein	4440588-4440606	TTAGGTGAGCCTAACCTAA	2
	2SCD60.15		Putative antibiotic binding protein			
SCO5830	SC5B8.20c		Conserved hypothetical protein	6381358-6381376	AGAGGTGAGCCTAACCTGA	2
SCO5998-SCO5999	SCBAC1C11.01c	*murA2*	Putative UDP-N-acetylglucosamine transferase	6575219-6575237	CTAACTTAGCCTTACCTTA	This study
	SCBAC1C11.02	*sacA*	Aconitate hydratase			
SCO6158-SCO6159	SC1A9.22c		Hypothetical protein	6761465-6761483	AAAGGTAAGCCTTACCTTA	1
	SC1A9.23		Possible gntR family transcriptional regulator			
SCO6426	SC1A6.15		Uncharacterized protein	7095655-7095673	TCAGGTAAGCCTTACCTAA	1
SCO7400	SC10G8.28c	*cdtC*	Putative ABC-transport protein, ATP-binding component	8214157-8214175	GAAGGTTAGCCTAACCTAA	2

References:

1. Kim, Y., Roe, J.-H., Park, J.-H., Cho, Y.-J. & Lee, K.-L. Regulation of iron homeostasis by peroxide-sensitive CatR, a Fur-family regulator in Streptomyces coelicolor. *J. Microbiol.* **59**, 1083–1091 (2021).
2. Flores, F. J. & Martín, J. F. Iron-regulatory proteins DmdR1 and DmdR2 of Streptomyces coelicolor form two different DNA-protein complexes with iron boxes. *Biochem. J* **380**, 497–503 (2004).
