# Supplementary material for: Genome mining based on transcriptional regulatory networks uncovers a novel locus involved in desferrioxamine biosynthesis
Source: PLoS Biol. 2025 Jun 12;23(6):e3003183. doi: 10.1371/journal.pbio.3003183 (PMC12161575; doi:10.1371/journal.pbio.3003183)
Supplement: S3 Table — (PDF) [file pbio.3003183.s011.pdf]

Table S3. Overview of DFO producing strains and detected homologs of the *desG* and *desH* genes.

Genome ID	desGH present?	Capped present?	Species	Compounds	Reference
GCA_024505145.1	no	no	*Streptomyces rugosispiralis* strain RCU-064	E, E-OH	[1]
GCA_016775155.1	no	no	*Fulvivirga* sp. W222	fulvivirgamides	[2]
GCA_000091565.1	no	no	*Erwinia amylovora* CFBP 1430	D2, E, G, X	[3]
GCA_002878675.1	no	no	*Glutamicibacter arilaitensis* JB182	E	[4]
GCA_002890855.1	no	no	*Pseudomonas kunmingensis* CCUG 36651	D2, E, G, X	[5]
GCA_009295505.1	no	no	*Rouxiella chamberiensis* strain Arv20#4.1	E, G1	[6]
GCA_003989215.1	no	no	*Corynebacterium propinquum* HSID18034	dehydroxynocardamines	[7]
GCA_009769735.1	no	no	*Streptomyces* sp. BA2	E	[8]
GCA_011290345.1	no	no	*Streptomyces* sp. ID38640	E	[9]
GCA_003330865.1	yes	no	*Streptomyces atratus* SCSIOZH16	E	[10]
GCA_009600885.1	yes	no	*Streptomyces jumonjinensis* NRRL 5741	E	[11]
GCA_001013905.1	yes	no	*Streptomyces leeuwenhoekii* C34	E	[12]
GCA_000444875.1	yes	no	*Streptomyces collinus* Tü 365	E	[13]
GCA_003568625.1	Only DesG	yes	*Gordonia rubripertincta* CWB2	E, G1, X6, B, A1	[14]
GCA_005519465.1	yes	yes	*Streptomyces clavuligerus* ATCC 27064	E, B	[11]
GCA_001595505.1	yes	yes	*Micrococcus* sp. CH3	aryl	[15]
GCA_001595485.1	yes	yes	*Micrococcus* sp. CH7	aryl	[15]
GCA_000016425.1	yes	yes	*Salinispora tropica* CNB-440	A2, A1, A1b, B, N, D2, E, D1	[16]
GCA_003298855.1	yes	yes	*Micromonospora provocatoris* MT25	B	[17]
GCA_016803235.1	yes	yes	*Pimelobacter simplex* 3E	B, D3, E	[18]
GCA_001660045.1	yes	yes	*Streptomyces parvulus* 2297	E, B	[19]

GCA_015321285.1	yes	yes	*Streptomyces albidoflavus* ANT_B131	B, E	[20]
GCA_000495755.1	yes	yes	*Streptomyces albus* PVA94-07	B, glycoconjugates	[21]
GCA_001267885.1	yes	yes	*Streptomyces ambofaciens* ATCC 23877	B, E	[22]
GCA_900236505.1	yes	yes	*Streptomyces chartreusis* NRRL 3882	many	[23]
GCA_015767775.1	yes	yes	*Streptomyces clavuligerus* F1D7	B, E	[11]
GCA_000203835.1	yes	yes	*Streptomyces coelicolor* A3(2)		[24]
GCA_000349325.1	yes	yes	*Streptomyces davawensis* JCM 4913	B, unnamed derivatives	[25]
GCA_009600895.1	yes	yes	*Streptomyces katsurahamanus* T-272	B	[11]
GCA_000980885.2	yes	yes	*Streptomyces malaysiense* strain MUSC 136	B	[26]
GCA_014649835.1	yes	yes	*Streptomyces pilosus* JCM 4403	B	[27]
GCA_000154945.1	yes	yes	*Streptomyces pristinaespiralis* ATCC 25486	B, E	[28]
GCA_001865245.1	yes	yes	*Streptomyces* sp. CC53	B, E, aryl, propyl	[15]
GCA_001595515.1	yes	yes	*Streptomyces* sp. CC71	E, B	[15]
GCA_001865255.1	yes	yes	*Streptomyces* sp. CC77	B, E, aryl, propyl	[15]
GCA_000154965.1	yes	yes	*Streptomyces sviceus* ATCC 29083	B	[29]
GCA_008639165.1	yes	yes	*Streptomyces venezuelae* ATCC 10712	aryl, D1, B, propyl	[30]
GCA_008704515.1	yes	yes	*Streptomyces viridosporus* ATCC 39115	B, E	[31]
GCA_000698945.1	yes	yes	*Streptomyces wadayamensis* A23	B, E	[32]
GCA_000220705.2	yes	yes	*Streptomyces xinghaiensis* NRRL B-24674	B, A1	[33]
GCA_015680775.2	yes	yes	*Streptomyces* sp. BRB081	E, B, A1, D2	[34]
GCA_016432675.1	yes	yes	*Streptomyces* sp. I3(2020)	D1	[35]
GCA_016432685.1	yes	yes	*Streptomyces* sp. I4(2020)	D1	[35]
GCA_003725745.1	yes	yes	*Streptomyces* sp. I6	D1	[35]
GCA_003573595.1	yes	yes	*Streptomyces* sp. SHP22-7	D1	[35]

References:

1. Weeraphan, T. *et al.* Streptomyces rugosispiralis sp. nov., a Novel Actinobacterium Isolated from Peat Swamp Forest Soil That Produces Ansamycin Derivatives and Nocardamines. *Antibiotics (Basel)* **12**, (2023).
2. Wang, Z.-J. *et al.* Genome mining and biosynthesis of primary Amine-acylated desferrioxamines in a marine gliding bacterium. *Org. Lett.* **22**, 939–943 (2020).
3. Kachadourian, R. *et al.* Desferrioxamine-dependent iron transport in Erwinia amylovora CFBP1430: cloning of the gene encoding the ferrioxamine receptor FoxR. *Biometals* **9**, 143–150 (1996).
4. Aron, A. *et al.* Native electrospray-based metabolomics enables the detection of metal-binding compounds. *bioRxiv* (2019) doi:10.1101/824888.
5. Essén, S. A., Johnsson, A., Bylund, D., Pedersen, K. & Lundström, U. S. Siderophore production by Pseudomonas stutzeri under aerobic and anaerobic conditions. *Appl. Environ. Microbiol.* **73**, 5857–5864 (2007).
6. Proença, D. N. *et al.* Bacterial metabolites produced under iron limitation kill pinewood nematode and attract Caenorhabditis elegans. *Front. Microbiol.* **10**, 2166 (2019).
7. Stubbendieck, R. M. *et al.* Competition among nasal bacteria suggests a role for siderophore-mediated interactions in shaping the human nasal Microbiota. *Appl. Environ. Microbiol.* **85**, (2019).
8. Kum, E. & İnce, E. Genome-guided investigation of secondary metabolites produced by a potential new strain Streptomyces BA2 isolated from an endemic plant rhizosphere in Turkey. *Arch. Microbiol.* **203**, 2431–2438 (2021).
9. Sosio, M. *et al.* Analysis of the pseudouridimycin biosynthetic pathway provides insights into the formation of C-nucleoside antibiotics. *Cell Chem. Biol.* **25**, 540-549.e4 (2018).
10. Li, Y., Zhang, C., Liu, C., Ju, J. & Ma, J. Genome sequencing of Streptomyces atratus SCSIOZH16 and activation production of nocardamine via metabolic engineering. *Front. Microbiol.* **9**, (2018).
11. AbuSara, N. F. *et al.* Comparative genomics and metabolomics analyses of clavulanic acid-producing Streptomyces species provides insight into specialized metabolism. *Front. Microbiol.* **10**, 2550 (2019).
12. Rateb, M. E. *et al.* Diverse metabolic profiles of a Streptomyces strain isolated from a hyper-arid environment. *J. Nat. Prod.* **74**, 1965–1971 (2011).
13. Iftime, D. *et al.* Identification and activation of novel biosynthetic gene clusters by genome mining in the kirromycin producer *Streptomyces collinus* Tü 365. *J. Ind. Microbiol. Biotechnol.* **43**, 277–291 (2016).
14. Schwabe, R. *et al.* Cultivation dependent formation of siderophores by Gordonia rubripertincta CWB2. *Microbiol. Res.* **238**, 126481 (2020).
15. Cruz-Morales, P. *et al.* Actinobacteria phylogenomics, selective isolation from an iron oligotrophic environment and siderophore functional characterization, unveil new desferrioxamine traits. *FEMS Microbiol. Ecol.* **93**, (2017).
16. Ejje, N., Soe, C. Z., Gu, J. & Codd, R. The variable hydroxamic acid siderophore metabolome of the marine actinomycete Salinispora tropica CNB-440. *Metallomics* **5**, 1519–1528 (2013).

17. Abdel-Mageed, W. M. *et al.* Biotechnological and Ecological Potential of Micromonospora provocatoris sp. nov., a Gifted Strain Isolated from the Challenger Deep of the Mariana Trench. *Mar. Drugs* **19**, 243 (2021).
18. Hofmann, M. *et al.* Screening for microbial metal-chelating siderophores for the removal of metal ions from solutions. *Microorganisms* **9**, 111 (2021).
19. Gáll, T. *et al.* Optimization of desferrioxamine E production by Streptomyces parvulus. *Acta Microbiol. Immunol. Hung.* **63**, 475–489 (2016).
20. França, P. de *et al.* Genome mining reveals secondary metabolites of Antarctic bacterium Streptomyces albidoflavus ANT_B131 related to antimicrobial and antiproliferative activities. *Research Square* (2020) doi:10.21203/rs.3.rs-123886/v1.
21. Sekurova, O. N. *et al.* New deferoxamine glycoconjugates produced upon Overexpression of pathway-specific regulatory gene in the marine sponge-derived Streptomyces albus PVA94-07. *Molecules* **21**, 1131 (2016).
22. Barona-Gómez, F. *et al.* Multiple biosynthetic and uptake systems mediate siderophore-dependent iron acquisition in Streptomyces coelicolor A3(2) and Streptomyces ambofaciens ATCC 23877. *Microbiology* **152**, 3355–3366 (2006).
23. Senges, C. H. R. *et al.* The secreted metabolome of Streptomyces chartreusis and implications for bacterial chemistry. *Proc. Natl. Acad. Sci. U. S. A.* **115**, 2490–2495 (2018).
24. Sidebottom, A. M., Johnson, A. R., Karty, J. A., Trader, D. J. & Carlson, E. E. Integrated metabolomics approach facilitates discovery of an unpredicted natural product suite from Streptomyces coelicolor M145. *ACS Chem. Biol.* **8**, 2009–2016 (2013).
25. Hagihara, R., Katsuyama, Y., Sugai, Y., Onaka, H. & Ohnishi, Y. Novel desferrioxamine derivatives synthesized using the secondary metabolism-specific nitrous acid biosynthetic pathway in Streptomyces davawensis. *J. Antibiot. (Tokyo)* **71**, 911–919 (2018).
26. Ser, H.-L. *et al.* Streptomyces malaysiense sp. nov.: A novel Malaysian mangrove soil actinobacterium with antioxidative activity and cytotoxic potential against human cancer cell lines. *Sci. Rep.* **6**, 24247 (2016).
27. Günter, K., Toupet, C. & Schupp, T. Characterization of an iron-regulated promoter involved in desferrioxamine B synthesis in Streptomyces pilosus: repressor-binding site and homology to the diphtheria toxin gene promoter. *J. Bacteriol.* **175**, 3295–3302 (1993).
28. Craney, A., Ozimok, C., Pimentel-Elardo, S. M., Capretta, A. & Nodwell, J. R. Chemical perturbation of secondary metabolism demonstrates important links to primary metabolism. *Chem. Biol.* **19**, 1020–1027 (2012).
29. Giddings, L.-A. *et al.* Characterization of a broadly specific cadaverine N-hydroxylase involved in desferrioxamine B biosynthesis in Streptomyces sviceus. *PLoS One* **16**, e0248385 (2021).
30. Jones, S. E. *et al.* Streptomyces volatile compounds influence exploration and microbial community dynamics by altering iron availability. *MBio* **10**, (2019).
31. Imbert, M., Béchet, M. & Blondeau, R. Comparison of the main siderophores produced by some species of Streptomyces. *Curr. Microbiol.* **31**, 129–133 (1995).
32. Angolini, C. F. F. *et al.* Genome mining of endophytic Streptomyces wadayamensis reveals high antibiotic production capability. *J. Braz. Chem. Soc.* (2016) doi:10.5935/0103-5053.20160180.

33. Chen, L.-Y. *et al.* Genome mining of Streptomyces xinghaiensis NRRL B-24674T for the discovery of the gene cluster involved in anticomplement activities and detection of novel xiamycin analogs. *Appl. Microbiol. Biotechnol.* **102**, 9549–9562 (2018).
34. Tangerina, M. M. P. *et al.* Metabolomic study of marine Streptomyces sp.: Secondary metabolites and the production of potential anticancer compounds. *PLoS One* **15**, e0244385 (2020).
35. Handayani, I. *et al.* Mining Indonesian microbial biodiversity for novel natural compounds by a combined genome mining and molecular networking approach. *Mar. Drugs* **19**, 316 (2021).
