# Supplementary material for: Genome mining based on transcriptional regulatory networks uncovers a novel locus involved in desferrioxamine biosynthesis
Source: PLoS Biol. 2025 Jun 12;23(6):e3003183. doi: 10.1371/journal.pbio.3003183 (PMC12161575; doi:10.1371/journal.pbio.3003183)
Supplement: S4 Table — (PDF) [file pbio.3003183.s012.pdf]

Table S4. DFO-related metabolites (*m/z* tolerance 0.001 or 5 ppm).

**DFO type**	**B M+H**	**B +Fe-2H**	**B +Al**	**E +Na**	**E M+H**	**E +Fe-2H**	**E +Al**	**E +Na**
Predicted	561.3606	614.2738	585.3187	583.3426	601.3556	654.2688	625.3137	623.3376
SCO4048	561.3610		585.3197		601.3555			623.3375
SCO4049	561.3610	614.2727	585.3197		601.3555	654.2678	625.3141	623.3375
SCO4050	561.3610	614.2727	585.3197		601.3555	654.2678	625.3141	623.3375
