# Supplementary material for: Genome mining based on transcriptional regulatory networks uncovers a novel locus involved in desferrioxamine biosynthesis
Source: PLoS Biol. 2025 Jun 12;23(6):e3003183. doi: 10.1371/journal.pbio.3003183 (PMC12161575; doi:10.1371/journal.pbio.3003183)
Supplement: S5 Table — (PDF) [file pbio.3003183.s013.pdf]

Table S5. Overview of the plasmids and constructs.

Name	Description	Reference
pCRISPR-cBEST	C to T base editor for actinomycetes, containing sgRNA cassette, Streptomyces codon optimized spCas9n (D10A)-rAPOBEC1 (cysteine deaminase)- Uracil Glycosylase Inhibitor (UGI) and sgRNA casette	1
pGWS1370	pSET152 containing an sgRNA scaffold and Pgapdh-dCas9	2
pGWS1384	As pCRISPR-cBEST but spCas9n (D10A)-rAPOBEC1(cystein deaminase)-Uracil Glycosylase Inhibitor (UGI) fusion protein was expressed under the control of Pgapah	This study
pGWS1582	pGWS1384 with spacer targeting SCO4048 for W55* inserted.	This study
pGWS1584	pGWS1384 with spacer targeting SCO4048 for Q68* inserted.	This study
pGWS1585	pGWS1384 with spacer targeting SCO4049 for W61* inserted.	This study
pGWS1589	pGWS1384 with spacer targeting SCO4050 for W43* inserted.	This study
pGWS1590	pGWS1384 with spacer targeting SCO4050 for Q91* inserted.	This study
pSET152	E. coli/Streptomyces shuttle vector, integrative in the ΦC31 attachment site in *Streptomyces*	3
pGWS1586	pSET152 with +1/+724 region of SCO4048 under the control of gap promoter of *S. coelicolor*	This study
pGWS1597	pSET152 with +1/+2347 SCO4049 under the control of gap promoter of *S. coelicolor*	This study
pGWS1598	pSET152 with +1/+619 SCO4050 under the control of gap promoter of *S. coelicolor*	This study

References:

1. Tong, Y. *et al.* Highly efficient DSB-free base editing for streptomycetes with CRISPR-BEST. *Proc. Natl. Acad. Sci. U. S. A.* **116**, 20366–20375 (2019).
2. Zhang, L. *et al.* An Alternative and Conserved Cell Wall Enzyme That Can Substitute for the Lipid II Synthase MurG. *MBio* **12**, (2021).
3. Bierman, M. *et al.* Plasmid cloning vectors for the conjugal transfer of DNA from Escherichia coli to Streptomyces spp. *Gene* **116**, 43–49 (1992).
