# Supplementary material for: Genome mining based on transcriptional regulatory networks uncovers a novel locus involved in desferrioxamine biosynthesis
Source: PLoS Biol. 2025 Jun 12;23(6):e3003183. doi: 10.1371/journal.pbio.3003183 (PMC12161575; doi:10.1371/journal.pbio.3003183)
Supplement: S6 Table — (PDF) [file pbio.3003183.s014.pdf]

Table S6. Overview of the oligonucleotides.

Name	5'-3'sequence #	Description
SCO4048_W55	CGGTTGGTAGGATCGACGGCGCCGCACCCACATCGTGGGGTTTTAGAGCTAGAAATAGC	For spacer insertion using single strand DNA(ssDNA) bridging method
SCO4048_Q68b	CGGTTGGTAGGATCGACGGCGCACCAGCGGGGGTACACGCGTTTTAGAGCTAGAAATAGC	
SCO4049_W61	CGGTTGGTAGGATCGACGGCCGGTCCCAGGACAGGGCCTCGTTTTAGAGCTAGAAATAGC	
SCO4050_W43	CGGTTGGTAGGATCGACGGCCACCCAGCCGTGGACCACCGGTTTTAGAGCTAGAAATAGC	
SCO4050_Q91	CGGTTGGTAGGATCGACGGCGCTCCAGACCTACGATCCCGGTTTTAGAGCTAGAAATAGC	
sg_T7_R_SnaBI	CAGTGGTTATGCTAGTTACGCCTACGTA	For validation of spacer insertion
SCO4048_check_F	GCTCACCTAAGTTATGGCGGTCG	For the amplification of sequencing fragments derived from corresponding mutants
SCO4048_check_R	GAGTCGGTACGAAACGGCAGG	
SCO4049_check_F	CGACCGCCATAACTTAGGTGAGCC	
SCO4049_check_R	GACGTCGAACTCCGGGCAGGA	
SCO4050_check_F	GGACCACGACTGCGTCCTGTG	
SCO4050_check_R	TGCTTGAGGAAGCGGGCGATC	
Pgap_F	AGGAAACAGCTATGACATGATTACG**GAATTC**GTCCTCGCCGACGAGGCCTC	For amplification of gap promoter region
Pgap_R	GAACCGATCTCCTCGTTGGTACGCCG	
sco4048_F	TACCAACGAGGAGATCGGTTATGGGGCAGGGGCGGGGCTGG	For amplification of coding regions of corresponding genes
sco4048_R	GCTTGGGCTGCAGGTCGACT**TCTAGA**TTAGGTCGCGCGCCAGTACCCCAGCG	
sco4049_F	TACCAACGAGGAGATCGGTTGTGAGCGCCGAGACCTACCGC	
sco4049_R	GCTTGGGCTGCAGGTCGACT**TCTAGA**TTAGTCATCGGATTCCTTCGTGAGCCGG	
sco4050_F	TACCAACGAGGAGATCGGTTATGACTGACGCACCCGCGACC	
sco4050_R	GCTTGGGCTGCAGGTCGACT**TCTAGA**TTACGCGCCGAACGCTACCTCC	

Restriction sites used for cloning are underlined and in bold face. GAATTC, EcoRI; TCTAGA, XbaI.
