# Supplementary material for: Genome mining based on transcriptional regulatory networks uncovers a novel locus involved in desferrioxamine biosynthesis
Source: PLoS Biol. 2025 Jun 12;23(6):e3003183. doi: 10.1371/journal.pbio.3003183 (PMC12161575; doi:10.1371/journal.pbio.3003183)
Supplement: S7 Table — (PDF) [file pbio.3003183.s015.pdf]

Table S7. Desferrioxamine gene cluster of *Gordonia rubripertincta* CWB2.

**Locus tag**	**Predicted function**	**Gene ID**	**Low-Fe/High-Fe expression ratio**
GCWB2_02925	N-acetyltransferase		1.5589
GCWB2_02930	Salicylate - AMP ligase	MbtA	3.2547
GCWB2_02935	Salicylate synthase	MbtI	3.6229
GCWB2_02940	Acyl carrier protein	MbtL	3.1964
GCWB2_02945	Acyl-[acyl carrier protein] dehydrogenase	MbtN	1.5481
GCWB2_02950	Putative diacylglycerol O-acyltransferase		1.7149
GCWB2_02955	Hypothetical protein		1.7913
GCWB2_02960	Siderophore binding protein		0.559
GCWB2_02965	Lysine N-monooxygenase	DesB	6.4957
GCWB2_02970	Lysine decarboxylase	DesA	7.5572
GCWB2_02975	Condensation domain		7.3817
GCWB2_02980	Penicillin acylase	DesG	6.7326
GCWB2_02985	Desferrioxamine ligase	DesD	13.372
GCWB2_02990	Desferrioxamine acyltransferase	DesC	21.28
GCWB2_02995	NRPS	MbtB	27.081
GCWB2_03000	Fatty acid - AMP ligase	MbtM	21.01
GCWB2_03005	ABC importer	IrtA	11.221
